# Red and far-red light improve the antagonistic ability of *Trichoderma guizhouense* against phytopathogenic fungi by promoting phytochrome-dependent aerial hyphal growth

**DOI:** 10.1371/journal.pgen.1011282

**Published:** 2024-05-20

**Authors:** Tingting Sun, Yifan Li, Jie Li, Jia Gao, Jian Zhang, Reinhard Fischer, Qirong Shen, Zhenzhong Yu

**Affiliations:** 1 Jiangsu Provincial Key Lab for Organic Solid Waste Utilization, Key Lab of Organic-based Fertilizers of China, Jiangsu Collaborative Innovation Center for Solid Organic Waste Resource Utilization, Educational Ministry Engineering Center of Resource-saving fertilizers, Nanjing Agricultural University, Nanjing, China; 2 Department of Microbiology, Institute for Applied Biosciences, Karlsruhe Institute of Technology (KIT) - South Campus, Karlsruhe, Germany; CAU: Christian-Albrechts-Universitat zu Kiel, GERMANY

## Abstract

Light as a source of information regulates morphological and physiological processes of fungi, including development, primary and secondary metabolism, or the circadian rhythm. Light signaling in fungi depends on photoreceptors and downstream components that amplify the signal to govern the expression of an array of genes. Here, we investigated the effects of red and far-red light in the mycoparasite *Trichoderma guizhouense* on its mycoparasitic potential. We show that the invasion strategy of *T*. *guizhouense* depends on the attacked species and that red and far-red light increased aerial hyphal growth and led to faster overgrowth or invasion of the colonies. Molecular experiments and transcriptome analyses revealed that red and far-red light are sensed by phytochrome FPH1 and further transmitted by the downstream MAPK HOG pathway and the bZIP transcription factor ATF1. Overexpression of the red- and far-red light-induced *fluffy* gene *fluG* in the dark resulted in abundant aerial hyphae formation and thereby improvement of its antagonistic ability against phytopathogenic fungi. Hence, light-induced *fluG* expression is important for the mycoparasitic interaction. The increased aggressiveness of *fluG*-overexpressing strains was phenocopied by four random mutants obtained after UV mutagenesis. Therefore, aerial hyphae formation appears to be a trait for the antagonistic potential of *T*. *guizhouense*.

## Author summary

Light controls a large proportion of the genome. While *Trichoderma* species encode the red-light receptor phytochrome, a detailed study of red-light responses and signaling was lacking. Here, we show that the mycoparasitic fungus *Trichoderma guizhouense* uses distinct strategies to attack phytopathogenic fungi. It can overgrow or invade the colonies of the attacked species. Red and far-red light promoted aerial hyphal growth and thereby increased both, overgrowth or invasion of the host colonies. The effect required the phytochrome-signaling pathway. We further show that screening for mutants producing more aerial hyphae proves to be an easy way to develop strains with improved biocontrol performance.

## Introduction

Incident light is a crucial signal for life activities of microorganisms [1–3]. It regulates asexual and sexual development, primary and secondary metabolism, the circadian clock and phototropism of fungi, as well as surface attachment and biofilm formation of bacteria [4–5]. As the dominant degraders of biomass in terrestrial ecosystems, fungi have evolved complex mechanisms to deal with a variety of environmental signals [6,7]. So far, great efforts have been made to understand light responses and signaling in several fungal species with respect to different phenomena. However, fungi living in natural habitats, besides responding to light and other abiotic environmental signals, continually communicate or interact with other organisms. How light affects these communication or interaction processes including interspecies antagonism of fungi remains to be elucidated.

Fungi, equipped with photoreceptors of different kinds, potentially respond to different wavelengths such as blue, red, far-red, green and UV light [2,8]. Light signaling and responses have been well studied in the model filamentous fungi *Neurospora crassa* and *Aspergillus nidulans*. In *A*. *nidulans*, red light signal is decisive for the balance between asexual and sexual development, which is sensed by the red-light receptor phytochrome (FphA) and further integrated into the mitogen-activated protein kinase (MAPK) HOG (SAK) pathway [9–13]. Likewise, in the phytopathogenic fungus *Alternaria alternata* and the entomopathogenic fungus *Beauveria bassiana* phytochrome participates in red light signaling and the regulation of asexual development [14,15].

Vegetative hyphae of fungi grow from the substrate into the air to form aerial hyphae, which, stimulated by a series of intra- and extracellular signals, may differentiate into conidiophores producing asexual conidia [16–18]. This process and the dispersal of mature spores are of great importance for fungal survival under unfavorable conditions. The growth of aerial hyphae is genetically controlled by an upstream developmental activation (UDA) pathway, consisting of the core component FluG and other essential proteins, FluA-FluE encoded by the *fluffy* genes [18,19]. The *fluffy* gene *fluG* is highly expressed in vegetative hyphae and responds to intracellular stimuli [19]. In *A*. *nidulans*, the expression of *fluG* and the other *fluffy* genes are induced by light and lack of FluG results in a *fluffy* phenotype and a defect in conidiation [20–22].

*Trichoderma* species are efficient antagonists against phytopathogenic fungi and are plant growth promoters and have thus been widely used in agriculture [23,24]. Their antagonistic ability is attributed to the direct mycoparasitic behavior involving secreted cell wall degrading enzymes and antifungal metabolites, and an aggressive mode of growth to compete for nutrients and space, which secure their leading roles in interspecies interactions [24,25]. Early studies showed that the mycoparasitic fungus *Trichoderma guizhouense* is antagonistic against different phytopathogenic fungi [26–28].

Here, we investigated how red and far-red light affect the interspecies antagonism between *Trichoderma* and phytopathogenic fungi. In red and far-red light, *T*. *guizhouense* produced more aerial hyphae, which helped to more quickly penetrate or overgrow the colonies of the phytopathogenic fungi. Our data indicate that red and far-red light were sensed by phytochrome FPH1 and further transmitted by the MAKP HOG pathway and the transcription factor ATF1 to control aerial hyphal growth and conidiation. This study demonstrates the importance of the light in fungal-fungal interactions.

## Results

### The *T*. *guizhouense* invasion strategy depends on the attacked species

**Fig 1:**
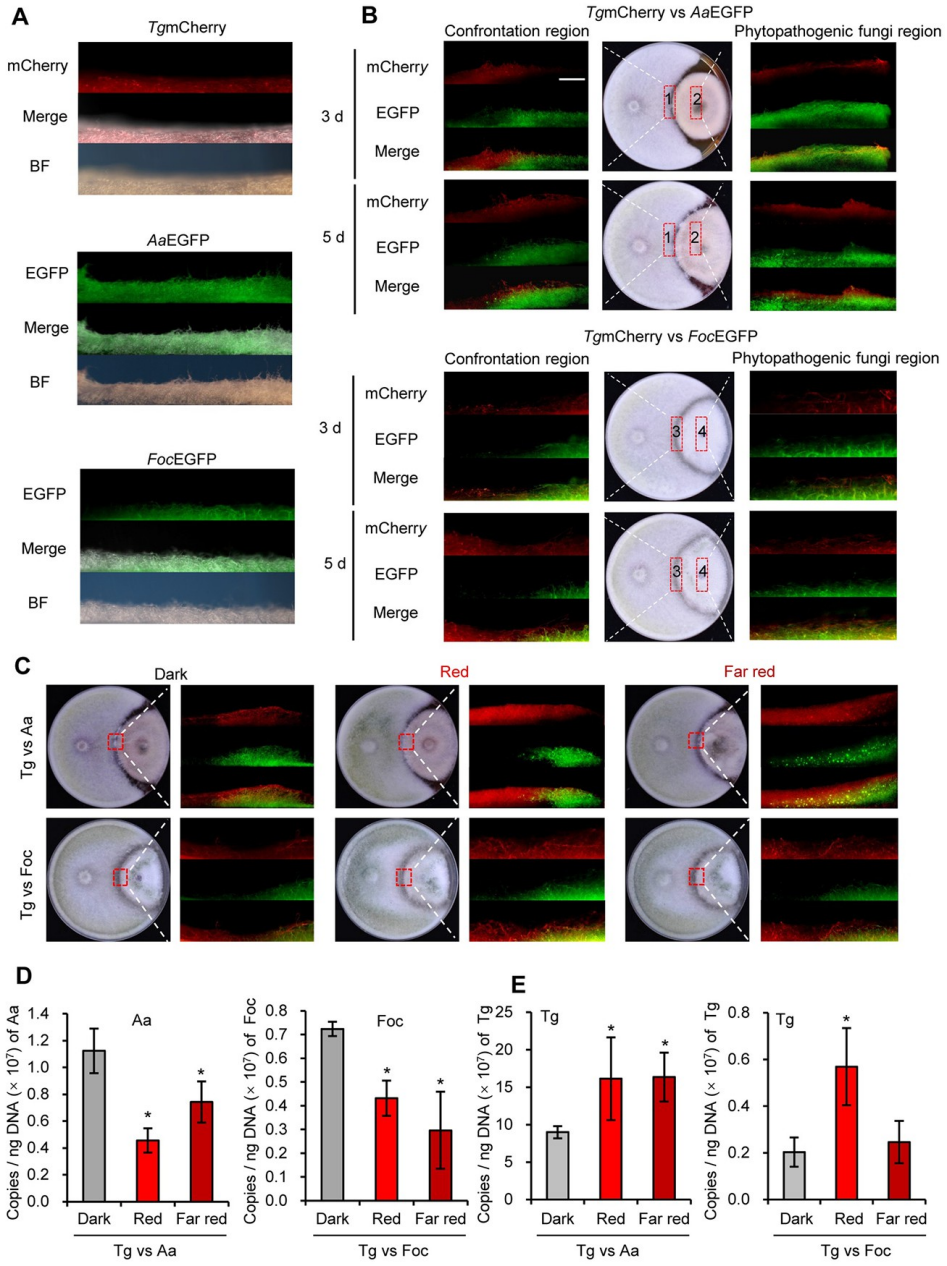
Dual confrontation assays between *T*. *guizhouense* and the plant pathogenic fungi. (A). Fluorescent images of mCherry-labelled *T*. *guizhouense* (*Tg*mCherry) and EGFP-labelled phytopathogens *A*. *alternata* (*Aa*EGFP) and *F*. *oxysporum* (*Foc*EGFP). Strains were cultured for 3 days at 28°C in the dark. (B). Dual confrontation assays between *T*. *guizhouense* and *A*. *alternata* or *F*. *oxysporum* in the dark. Strains were co-cultured for 3 or 5 days at 28°C in the dark. Vertical sections of the areas indicated in red boxes were zoomed in under a stereo microscope. Scale bar, 1 mm. (C). Dual confrontation assays between *T*. *guizhouense* and *A*. *alternata* or *F*. *oxysporum* under red and far-red light conditions. Strains were co-cultured for 5 days at 28°C in the dark or in constant red or far-red light. Vertical sections of the areas indicated in red boxes were zoomed in under a stereo microscope. Scale bar, 1 mm. (D). Detection of the *A*. *alternata* or *F*. *oxysporum* DNA copies in the colonies of the pathogens in dual confrontation assays. DNA of the mycelia of the whole phytopathogen colonies was extracted after 5 days of incubation under different light conditions. Aa: *A*. *alternata*, Foc: *F*. *oxysporum*, Tg: *T*.*guizhouense*. (E). Detection of the *T*. *guizhouense* DNA copies in the colonies of the pathogens. The error bar represents the standard deviation (SD) of three biological replicates. Statistically significant differences were evaluated by one-way analysis of variance (ANOVA) comparison.

*T*. *guizhouense* exhibits a broad spectrum of antagonistic activity against phytopathogenic fungi [27]. To visualize the colonizing process more clearly during the interspecies interaction, we labelled *T*. *guizhouense* wild type strain with mCherry (*Tg*mCherry) that was constitutively expressed under the control of the *Trichoderma reesei cdna1* promoter, and the phytopathogenic fungi *A*. *alternata* and *F*. *oxysporum* were labelled with EGFP that was under the control of the *T*. *reesei tef1* promoter (Fig 1A). The mCherry-labelled *T*. *guizhouense* strain was then confronted with EGFP-labelled *A*. *alternata* and *F*. *oxysporum* separately in the dark. After 3 days of confrontation, the red aerial hyphae of *T*. *guizhouense* began to overgrow the green mycelia of *A*. *alternata* (Fig 1B). After 5 days, the red mycelia had covered the whole green colony. Notably, the red mycelia were limited to the colony surface of *A*. *alternata*, and no red fluorescence signals were detected in the colony. Hence, it may feed on the mycelia of *A*. *alternata* (mycoparasitism) and competes for space of growth, instead of competing for nutrients from the substrate. However, confronted with *F*. *oxysporum*, the hyphae of *T*. *guizhouense* grew along the substrate surface and had penetrated the *F*. *oxysporum* colony after 3 days, indicating that *T*. *guizhouense* competed for nutrients with *F*. *oxysporum* during the confrontation. Therefore, *T*. *guizhouense* colonizes *F*. *oxysporum* and *A*. *alternata* in different ways. It likely attacks *A*. *alternata* in a direct mycoparasitic manner, and yet, when confronted with *F*. *oxysporum*, it competes first for the nutrients from substrate.

### The antagonistic activity of *T*. *guizhouense* against phytophathogenic fungi is improved under red and far-red light conditions

To evaluate the effect of light on this interspecies interaction, *T*. *guizhouense* was confronted with *A*. *alternata* and *F*. *oxysporum* for 5 days under light and dark conditions and the vertical sections of contact areas of two colonies were observed regularly under the microscope for 5 days (Fig 1C). Under the red and far-red light conditions, more aerial hyphae of *T*. *guizhouense* appeared and formed denser networks on top of the *A*. *alternata* colonies than in the dark, indicating that red and far-red light facilitated the overgrowing process. After confronted with *F*. *oxysporum* in the dark for 5 days, the *T*. *guizhouense* strain invaded the colony along the substrate surface. More red aerial hyphae were observed inside the colonies of *F*. *oxysporum* under red and far-red conditions than in the dark. Thus, the invasion was promoted by red and far-red light.

To quantify the levels of each fungal species, the DNA copies of each strain in the colonies being attacked was determined. Absolute quantitative PCR with strain-specific primer pairs for *T*. *guizhouense*, *A*. *alternata* and *F*. *oxysporum* were performed using the DNA samples extracted from the mycelia of the whole phytopathogen colonies as templates. In red and far-red light, less DNA copies of two phytopathogens were detected than in the dark, and more DNA copies of *T*. *guizhouense* in the *A*. *alternata* colonies in red and far-red light were detected than in the dark (Fig 1D and 1E). DNA copies of *T*. *guizhouense* in the *F*. *oxysporum* colonies were increased by red light. These results demonstrate that red and far-red light have a positive impact on the antagonistic ability of *T*. *guizhouense*.

### Red and far-red light promote aerial hyphal growth and conidiation of *T*. *guizhouense* through the FPH1 and the MAPK HOG pathway

**Fig 2:**
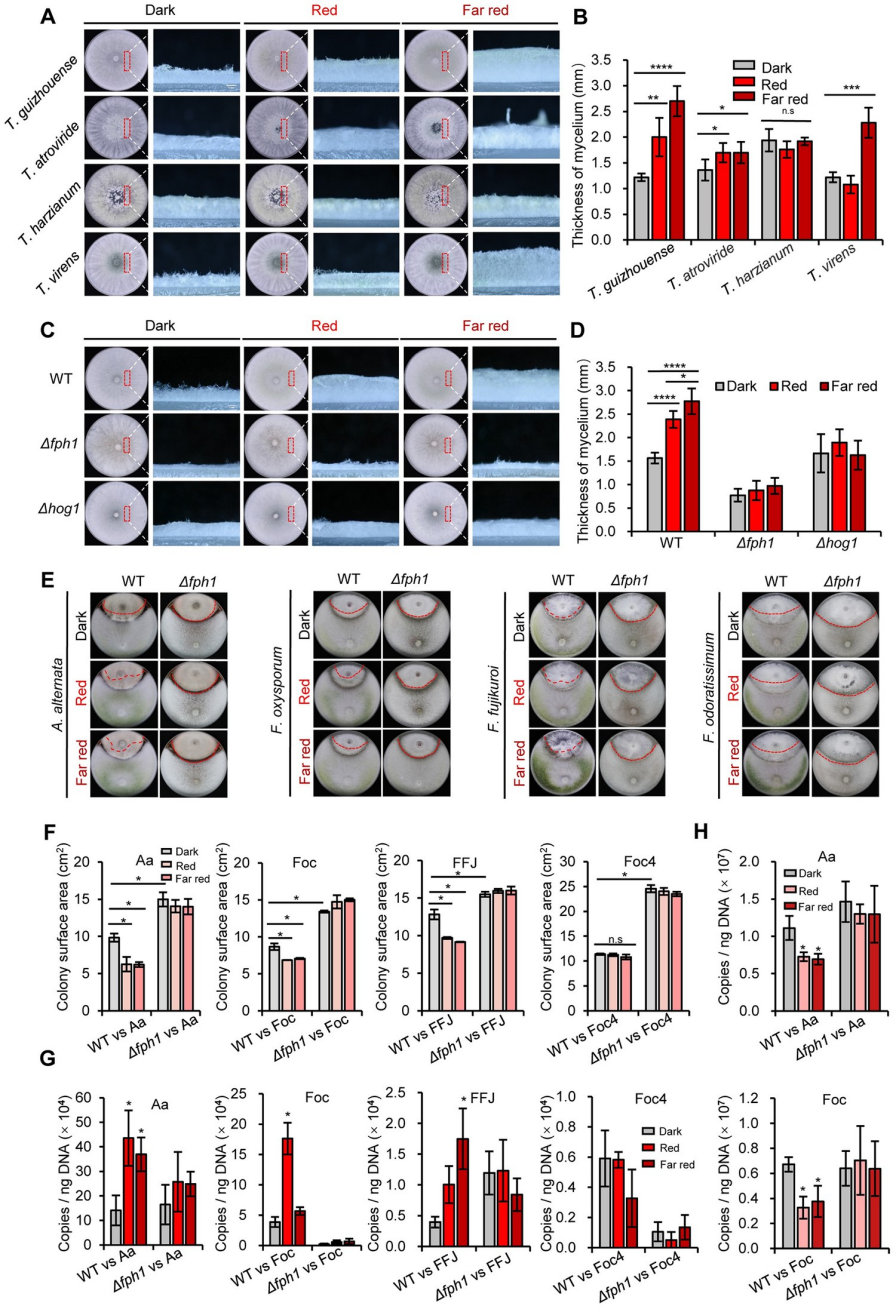
Aerial hyphal growth and antagonistic activity analyses of wild type and the *Δfph1* strain under red and far-red light conditions. (A). Phenotypes of aerial hyphae in four *Trichoderma* species. All strains were incubated in red and far-red light at 28°C for 3 days. Vertical sections of the colonies were observed and photographed under a stereoscope. Scale bar,1 mm. (B). Thickness measurement of aerial hyphae in different *Trichoderma* species grown in red and far-red light. Vertical sections of the colonies were photographed, and thickness of mycelia was determined using the software ImageJ. (C). Phenotypes of aerial hyphae in the wild type, the *Δfph1*, and the *Δhog1* strains. All strains were incubated as in (A). Scale bar, 1 mm. (D). Thickness measurement of aerial hyphae in wild type, the *Δfph1*, and the *Δhog1* strains. The hyphae were observed under a stereoscope. The experiment was repeated three times in five replicates for each strain. Thickness of the aerial hyphae was measured as above. Statistically significant differences were evaluated by Student’s *t*-test: * *p* < 0.05; ** *p* < 0.01; *** *p* < 0.001; **** *p* < 0.0001. (E). Antagonistic activity analysis of wild type and the *Δfph1* strain against four phytopathogenic fungi in red and far-red light. Plates were incubated at 28°C for 5 days under different light conditions. The red dotted line indicates the colony surface of pathogens that was not covered by *T*. *guizhouense*. (F). Areas of colony surface of four phytopathogens that were not covered by wild type and the *Δfph1* under different light light conditions. The areas were determined using ImageJ. (G). Detection of *T*. *guizhouense* DNA in the colonies of the pathogens in dual confrontation assays. *T*. *guizhouense* strains were confronted with the phytopathogenic fungi *A*. *alternata* (Aa), *F*. *oxysporum* (Foc), *F*. *fujikuroi* (FFJ) and *F*. *odoratissimum* (Foc4) under different light conditions at 28°C for 5 days. DNA of the mycelia of the whole phytopathogen colonies was extracted. The error bar represents the standard deviation (SD) of three biological replicates. Statistically significant differences were evaluated by Student’s *t*-test: * *p* < 0.05. (H). Detection of *A*. *alternata* or *F*. *oxysporum* DNA copies in the colonies of the pathogens confronted with the wild type and the *Δfph1* strains.

Next, we tested the impact of red and far-red light on the morphogenesis of four *Trichoderma* species capable of antagonizing phytopathogens. Intriguingly, red and far-red light promoted the growth of aerial hyphae significantly in *T*. *guizhouense* and *T*. *atroviride* (Fig 2A and 2B). In addition, while aerial hyphal growth of *T*. *virens* was promoted by far-red light, it did not respond to red light. The results further demonstrate that *Trichoderma* species potentially respond to red and far-red light, which moreover seem to be more sensitive to the latter.

Next, we performed a BLAST search against the *T*. *guizhouense* genome using the sequence of *A*. *nidulans* FphA as a query in the National Center for Biotechnology Information (NCBI) protein database. The protein OPB36558, designated as FPH1, has highest identity (52%, e-value: 2e-46) and similar domain arrangement with FphA (S1A Fig). To analyze if FPH1 is responsible for red and far-red light sensing, the *fph1* gene was deleted (*Δfph1*) as verified by Southern blot (S1B Fig). The complemented strain *fph1*^*C*^ was also constructed. In view of the involvement of the MAPK SakA (HogA) in red light signaling in *A*. *nidulans* [12], red and far-red light responses of the *Δhog1* strain, in which the MAPK HOG1 was absent, was also analyzed in the following study. The phenotypes of the wild type, the *Δfph1*, and the *Δhog1* strains were compared after 3 days of incubation. In contrast to wild type, the aerial hyphae of the *Δfph1*, and the *Δhog1* strains did not respond to red and far-red light (Fig 2C and 2D). The phenotype was rescued in the *fph1*^*C*^ strain (S2A and S2B Fig). Noteworthily, even in the dark, absence of phytochrome FPH1 resulted in attenuated aerial hyphal growth. This suggests that FPH1 not only controls red and far-red light promoted aerial hyphal growth but also maintain basal growth in the dark. Additionally, after 5 days, wild type both in red and far-red light produced yellow-green conidiophores, whereas in the *Δfph1*, and the *Δhog1* strains only a few were observed, indicating the involvement of FPH1 and HOG1 in conidiation. (S3A and S3B Fig).

### Phytochrome regulates the interspecies interactions between *T*. *guizhouense* and phytopathogenic fungi

We then hypothesized that the abundant aerial hyphae formed in red and far-red light contribute to the improved antagonistic activity. To test this, phytopathogenic fungi were confronted with the wild type and the *Δfph1* strains under different light conditions for 5 days. In the dark, the areas of colony surface of four phytopathogens that were not covered by wild type were smaller than those not covered by the *Δfph1* strain (Fig 2E and 2F). In red and far-red light, the areas of colony surface of *A*. *aternata* (Aa), *F*. *oxysporum* (Foc) and *F*. *fujikuroi* (FFJ) not covered by wild type were smaller than those in the dark, whereas the areas of colony surface of four phytopathogens not covered by the *Δfph1* strain were not affected by red and far-red light. The DNA copies of *T*. *guizhouense* in the colonies of pathogens were further measured. In comparison to dark condition, more DNA copies of *T*. *guizhouense* in red or far-red light were detected in the colonies of *A*. *alternata*, *F*. *oxysporum* and *F*. *fujikuroi* (Fig 2G). In the absence of FPH1, DNA copies were pronouncedly reduced under both dark and light conditions in the colonies of *F*. *oxysporum* and *F*. *odoratissimum*. We also measured the DNA copies of *A*. *alternata* and *F*. *oxysporum*. In red and far-red light, less DNA copies of them were detected in their colonies confronted with wild type than in the dark, and DNA copies of them were not affected by red and far-red light when confronted with the *Δfph1* strain (Fig 2H). Taken together, these results indicate that FPH1 is involved in the antagonistic activity of *T*. *guizhouense*, suggesting a novel role of FPH1 in the interspecies interactions.

### Red and far-red light activate the MAPK HOG (SAK) signaling pathway

**Fig 3:**
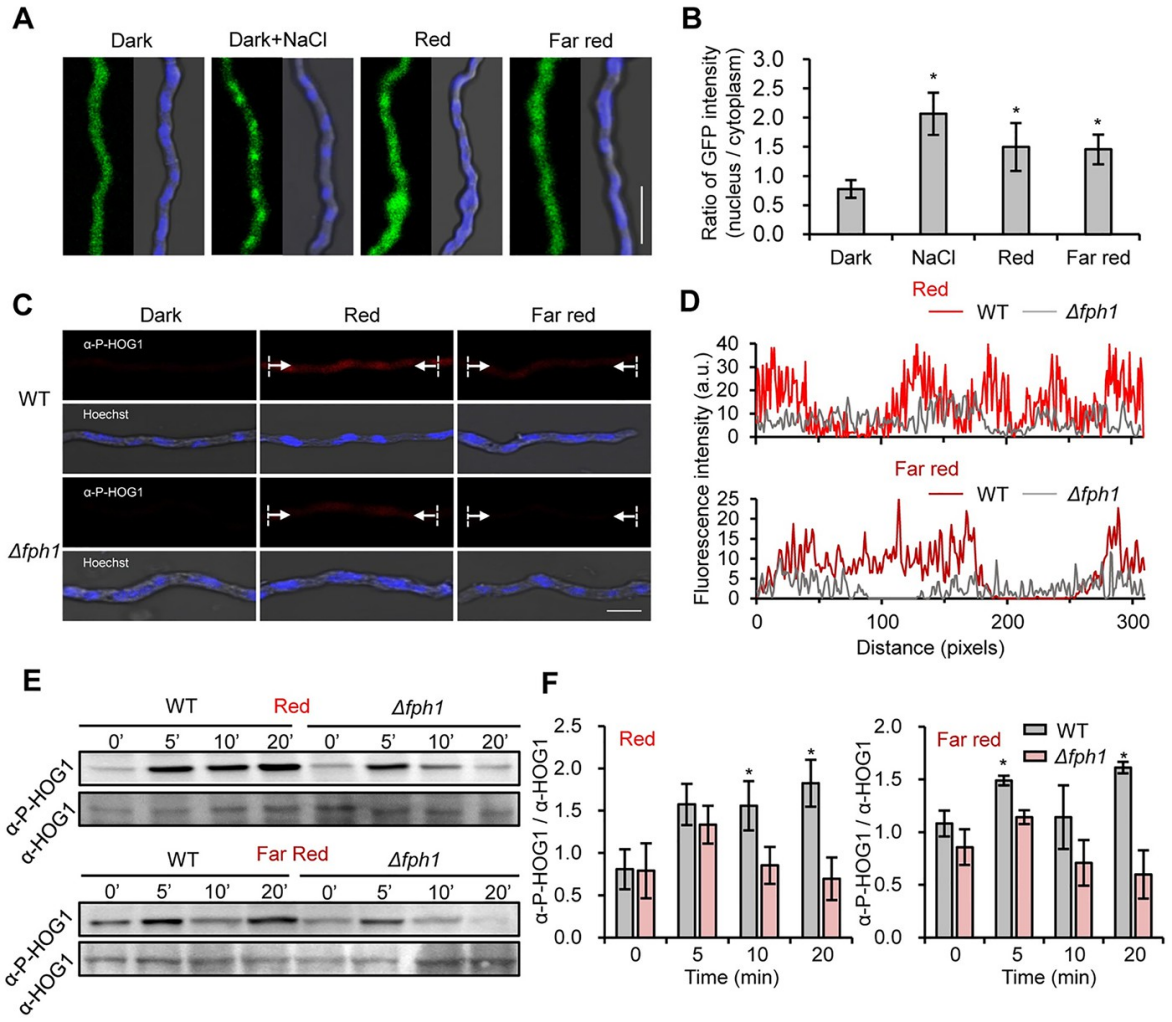
Subcellular localization and phosphorylation level of HOG1 upon red and far-red light exposure. (A). Subcellular localization of HOG1 in *T*. *guizhouense* after different treatments. Fresh conidia of a strain expressing HOG1-EGFP were inoculated on coverslips with 400 μl PDB medium and cultured in the dark at 28°C for 14 h. Afterwards, samples were exposed to red and far-red light or PDB medium containing 0.5 M NaCl in the dark for 5 min. The mycelium was then fixed with 4% formaldehyde for 10 min and washed with 1 × PBS before microscopy. Scale bar, 20 μm. (B). Quantification of EGFP intensity under each treatment. 10 cells were selected to calculate the ratio of EGFP intensity between the nucleus and the cytoplasm. The number represents the quantification of EGFP signals by ImageJ. The bars present mean values ± SD. (C). Immunofluorescence analysis of HOG1 phosphorylation in different strains. Conidia of the wild type and the *Δfph1* strains were incubated on coverslips with PDB for 14 h in the dark at 28°C and treated with red and far-red light or kept in the dark for 5 min before subjected to immunostaining. Nuclei were stained with Hoechst dye. Scale bar, 5 μm. (D). Fluorescence intensity profile (source: white arrow in (C)) of wild type treated with red light and far-red light. The number represents the quantification of red signals by ImageJ. (E). Western blot analysis of HOG1 phosphorylation under different light conditions. Each strain was cultured in PDB medium for 24 h and mycelia were harvested and frozen immediately after 5-, 10- and 20-min illumination with red or far-red light for protein extraction. 40 μg crude extract of each sample was used for Western blot. (F). Quantification of the relative phosphorylation level of HOG1 in (E).

Next, we checked the subcellular localization of HOG1 in *T*. *guizhouense* upon red and far-red light exposure. The strain expressing HOG1-EGFP was exposed to red and far-red light and salt stress (0.5 M NaCl) as a positive control. In the dark, EGFP signals were evenly distributed in nuclei and the cytoplasm, whereas after 5 min of red and far-red light exposure EGFP signals accumulated in nuclei (Fig 3A and 3B). To analyze the phosphorylation, immunostaining was performed with antibody against phosphorylated HOG1. Red fluorescent signals were observed in wild type after 5 min of illumination with red and far-red light. By contrast, in the *Δfph1* strain, signals of phosphorylated HOG1 were very weak (Fig 3C and 3D). Likewise, Western blot analysis showed the rapid phosphorylation of HOG1 in wild type and a reduced phosphorylation level in the *Δfph1* strain upon exposure to red and far-red light (Figs 3E, 3F, and S7). It is worth noting that both red and far-red light still caused HOG1 phosphorylation, albeit weakly, when FPH1 was absent.

### Genome-wide transcriptomic profiling reveals that far-red light controls more genes in the genome than red light

**Fig 4:**
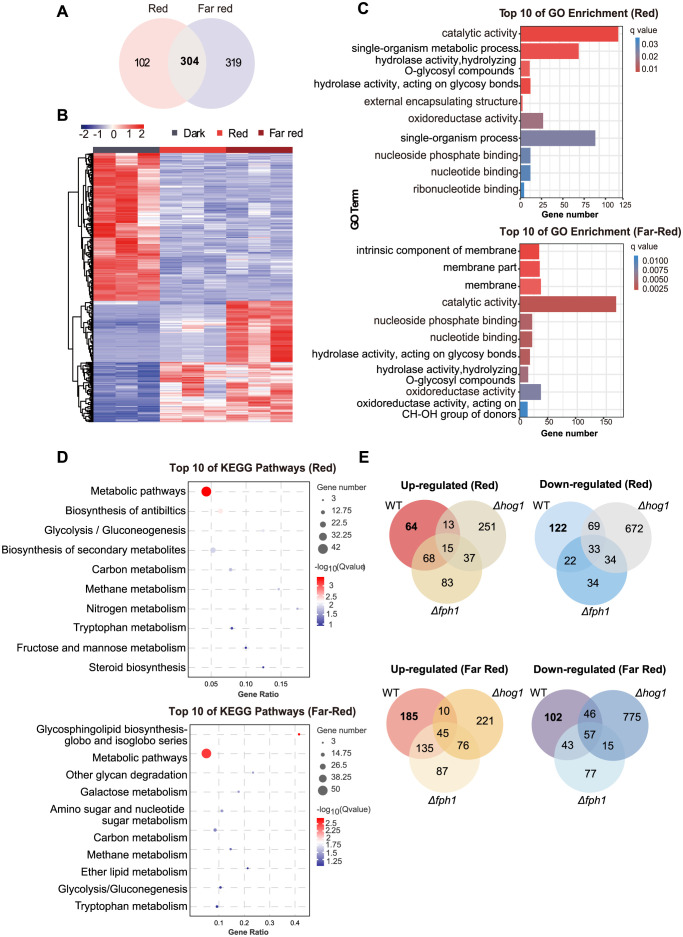
Transcriptome analysis of *T*. *guizhouense* exposed to red and far-red light. (A). Venn diagrams of the differentially expressed genes (DEGs) identified in the wild type strain treated with red and far-red light. (B). Heatmap of the transcript abundances of DEGs identified in the wild type. (C). Gene Ontology (GO) terms of DEGs identified in wild type. (D). Top 10 significantly enriched Kyoto Encyclopaedia of Genes and Genomes (KEGG) pathways of DEGs identified in wild type in response to red and far-red light. (E). Venn diagrams of DEGs identified in the wild type, the *Δfph1* and the *Δhog1* strains in response to red and far-red light.

To analyze the genome-wide gene expression in response to red and far-red light in *T*. *guizhouense*, we performed transcriptomic profiling of the wild type, the *Δfph1*, and the *Δhog1* strains. Samples for sequencing were treated for 45 min with red or far-red light after 24 h of culture in the dark. Differentially expressed genes (DEGs) (|log2 (fold change)| ≥ 1 and false discovery rate < 0.05) were identified and the reliability of transcriptome data was verified by measuring the expression level of a series of genes by RT-qPCR (S4A–S4D Fig). In total, 406 DEGs under red light treatment in wild type were identified, of which 160 genes were upregulated and 246 downregulated, whereas far-red light treatment resulted in 623 DEGs (375 up- and 248 down-regulated). 304 genes were co-regulated by red and far-red light (Fig 4A and 4B). The results demonstrate that gene expression is more sensitive to far-red light than to red light, consistent with the above result that thicker mycelia was formed in far-red light than in red light.

Next, the enrichment analysis of Gene Ontology (GO) terms and KEGG pathways was performed with these DEGs identified in red and far-red light. The top enriched GO term in red light was "catalytic activity", followed by "single-organism metabolic process" and "hydrolase activity". In far-red light, the top three enriched GO terms were "intrinsic component of membrane", "membrane part" and "membrane" (Fig 4C). 124 DEGs in red light and 167 in far-red light were enriched in the term of "catalytic activity", suggesting stronger impact of far-red light on the catalytic processes in the cells. KEGG pathway enrichment analysis revealed that the top three enriched pathways in red light were "metabolic pathways", "biosynthesis of antibiotics", and "glycolysis/gluconeogenesis" (Fig 4D). By contrast, in far-red light, the top enriched pathway was "glycosphingolipid biosynthesis-globo and isoglobo series", followed by "metabolic pathways" and "other glycan degradation".

### FPH1 and HOG1 are crucial for the transmission of red and far-red light signals

To gain insights into the regulatory networks of red and far-red light signaling in *T*. *guizhouense*, we further compared the DEGs identified in the *Δfph1* and the *Δhog1* strains with the ones identified in wild type. Surprisingly, absence of FPH1 did not abolish red and far-red light signaling, as there were still 362 and 574 genes differentially expressed in the *Δfph1* strain in response to red and far-red light, respectively. Venn diagrams showed 66.0% (268/406) of DEGs identified in wild type under red light condition and 55% (343/623) under far-red light condition were governed by FPH1, whereas 138 red and 280 far-red light regulated genes in wild type was independent of FPH1 (Fig 4E). This implies the existence of alternative mechanisms responsible for red and far-red light perception in *T*. *guizhouense*.

More surprisingly, more DEGs were identified in the *Δhog1* strain than in wild type. 1124 DEGs under red light condition were identified in the *Δhog1* strain, of which only 12.2% (138/1124) overlapped with the red light regulated genes in wild type. 67.9% (276/406) of red light regulated genes in wild type depended on HOG1. In far-red light, more DEGs (1284) were identified in the *Δhog1* strain and only 158 of them (12.3%) overlapped with the far-red light regulated genes identified in wild type. 74.6% (465/623) of far-red light regulated genes identified in wild type was controlled by HOG1. These results suggest that alternative pathway for red and far-red light signaling was strengthen after HOG pathway were shut down.

### The bZIP transcription factor *ATF1* regulates red and far-red light signaling and aerial hyphal growth in *T*. *guizhouense*

**Fig 5:**
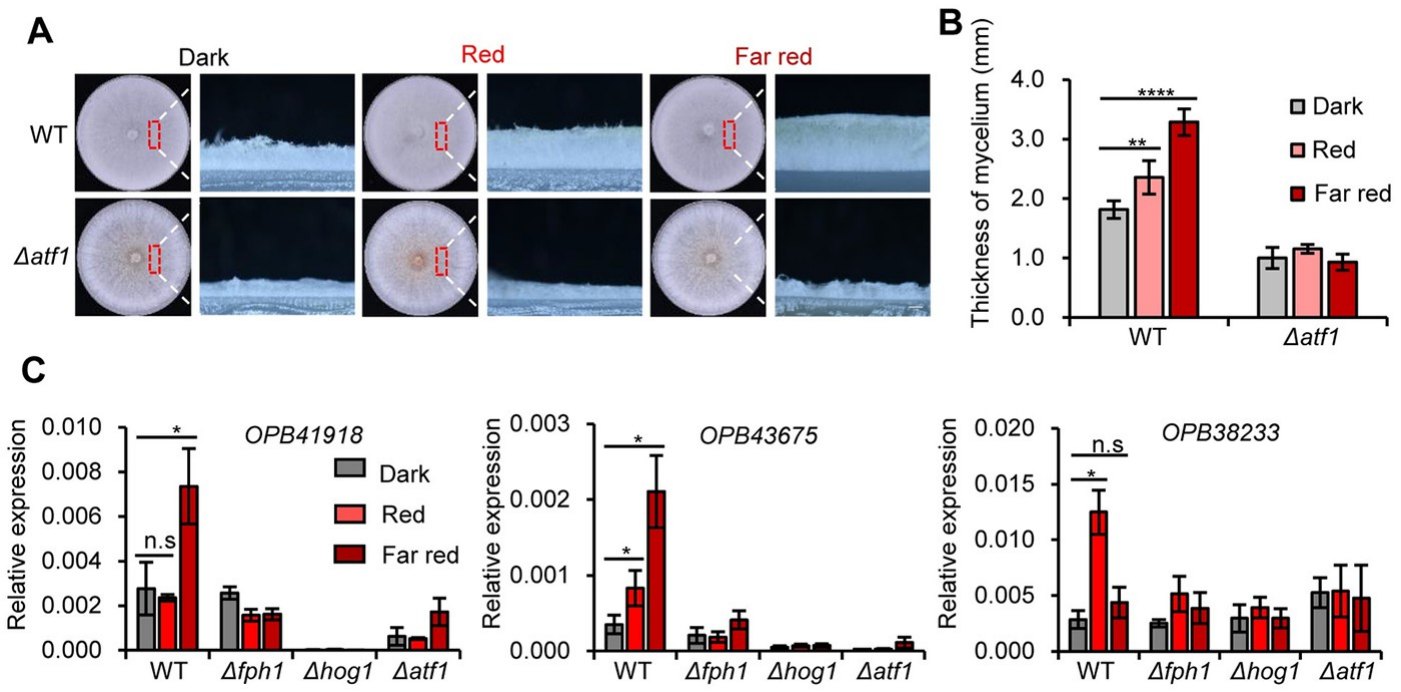
Aerial hyphal growth and gene expression analyses of the *Δatf1* strain. (A). Comparison of aerial hyphae growth in the wild type and the *Δatf1* strains. All strains were incubated on PDA plates at 28°C for three days under dark or light conditions. Vertical sections of the areas indicated in red boxes were zoomed in under a stereo microscope. Scale bar, 1 mm. (B). Thickness measurement of aerial hyphae in the wild type and *Δatf1* strains. The error bar represents the standard deviation (SD) of three biological replicates. Statistically significant difference is evaluated by Student’s *t*-test: * *p* < 0.05; ** *p* < 0.01; *** *p* < 0.001; **** *p* < 0.0001. (C). Expression levels of the genes induced by light in wild type, *Δfph1*, *Δhog1*, and *Δatf1* strains. All strains were illuminated with red or far-red light for 45 min. The expression level of each gene was normalized to the *tef1* gene. Error bars indicate the standard deviation. Statistically significant difference is evaluated by Student’s *t*-test: * *p* < 0.05; ** *p* < 0.01; *** *p* < 0.001; **** *p* < 0.0001.

In *A*. *nidulans*, the bZIP transcription factor AtfA is the downstream component of the HOG (SAK) pathway, which is responsible for sensing of oxidative stress [29]. We observed that in *T*. *guizhouense* lack of ATF1, the homolog of AtfA, reduced the aerial hyphal growth in the dark and the thickness of aerial hyphae decreased from 1.8 mm in wild type to 1.0 mm in the *Δatf1* strain (Fig 5A and 5B). To confirm if ATF1 is really involved in red and far-red light signaling, we chose three light-regulated genes OPB41918 (phosphatidyl synthase), OPB45957 (hypothetical protein), and OPB38233 (zinc binding oxidoreductase) randomly from DEGs identified above to measure their expression levels in different strains. Indeed, these genes did not respond to red and far-red light in the *Δfph1*, the *Δhog1*, and the *Δatf1* strains (Fig 5C). Therefore, FPH1, HOG1 and ATF1 coregulate the expression of these genes and the aerial hyphal growth.

### Overexpression of the *fluffy* gene *fluG* promotes aerial hyphal growth and increases the antagonistic ability of *T*. *guizhouense*

**Fig 6:**
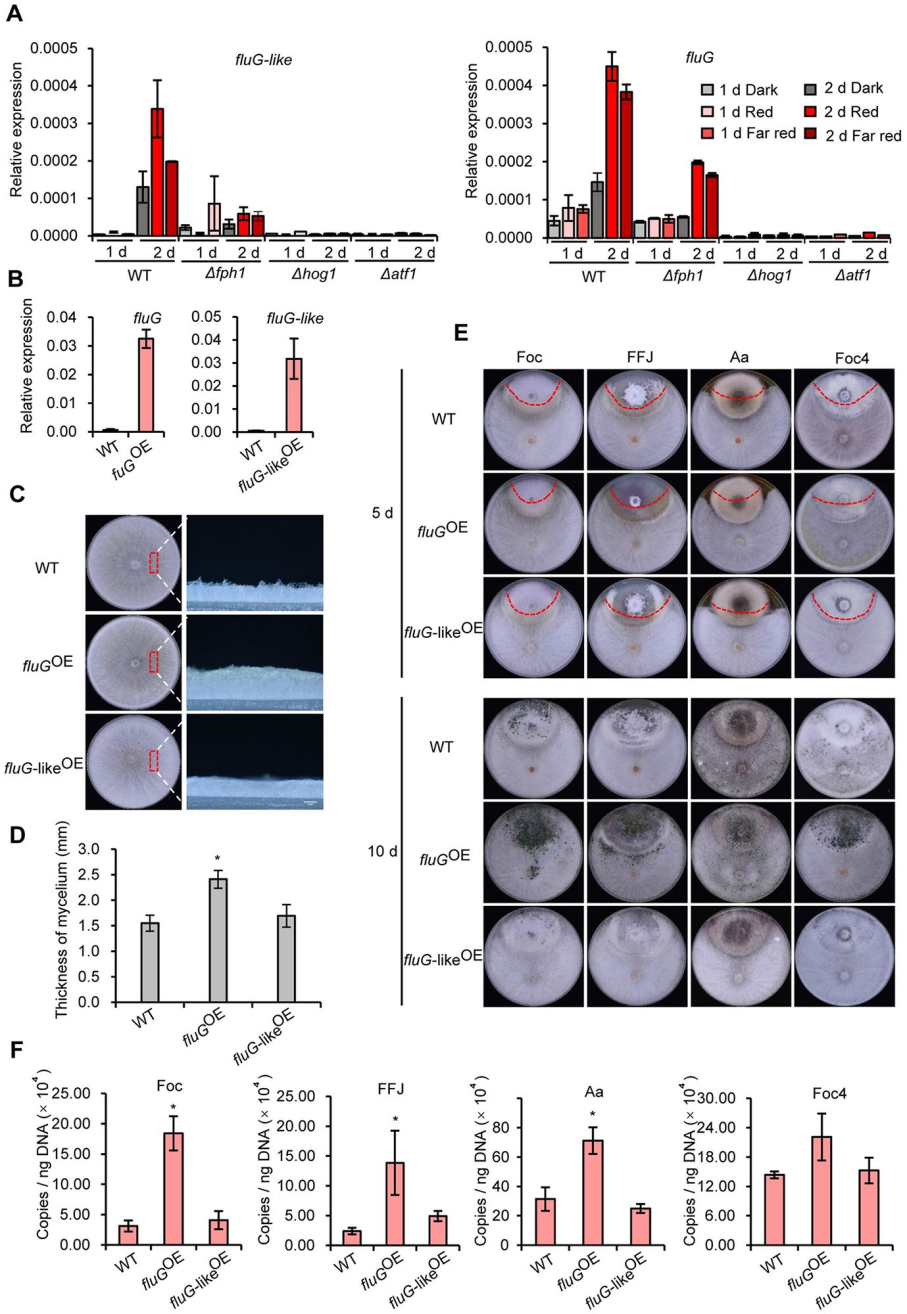
Aerial hyphal growth and antagonistic activity analyses of the *fluG*^*OE*^ and *fluG-like*^*OE*^ strains. (A). Expression levels of the *fluG* and *fluG-like* genes in the wild type, the *Δfph1*, *Δhog1* and the *Δatf1* strains under different light conditions. All strains were cultured in red or far-red light for 1 or 2 days. (B). Expression levels of the *fluG* and *fluG-*like genes in the *fluG*^OE^ and *fluG-*like^OE^ strains. The expression level of each gene was normalized to the *tef1* gene. Error bars indicate standard deviations. (C). Comparison of aerial hyphal growth in wild type, the *fluG*^OE^, and the *fluG-like*^OE^ strains. Strains were incubated on PDA plates at 28°C in the dark for 3 days. (D). Thickness measurement of the aerial hyphae in the *fluG*^OE^ and *fluG-like*^OE^ strains. The thickness of the aerial hyphae was determined by the software ImageJ. Error bar represents the standard deviation (SD) of five biological replicates. Significant differences between different strains with a single-factor random grouping model were statistically determined by one-way analysis of variance (ANOVA) comparison. (E). Antagonistic activity analysis of the wild type, *fluG*^OE^, and *fluG-*like^OE^ strains against *A*. *alternata*, *F*. *oxysporum*, *F*. *fujikuroi* and *F*. *odoratissimum*. Strains were incubated on PDA plates at 28°C in the dark for 5 or 10 days. Upper colony on the plate was the phytopathogen and lower was *T*. *guizhouense*. (F). Measurement of DNA copies of the wild type, *fluG*^OE^ and *fluG-*like^OE^ strains in the colonies of phytopathogenic fungi by absolute qPCR. *T*. *guizhouense* strains were confronted with the phytopathogenic fungi *A*. *alternata* (Aa), *F*. *oxysporum* (Foc), *F*. *fujikuroi* (FFJ) and *F*. *odoratissimum* (Foc4) in the dark at 28°C for 5 days.

In view of the foregoing results, we then hypothesized that abundant aerial hyphae formed in red and far-red light are closely associated with the improved antagonistic activity of *T*. *guizhouense* under red and far-red conditions. The genome of *T*. *guizhouense* encodes two homologs of the *A*. *nidulans* FluG (S5 Fig), which is the core regulator of aerial hyphal growth. We found that *fluG* and *fluG-*like were significantly induced especially after 2 days of culture in red and far-red light (Fig 6A). By contrast, they were not induced in the *Δhog1* and *Δatf1* strains. In the *Δfph1* strain, *fluG* was induced by red and far-red light but the expression levels were still significantly lower than those in wild type. To test our hypothesis, we overexpressed *fluG* (*fluG*^OE^) and *fluG*-like (*fluG-*like^OE^) under the control of *T*. *reesei cdna1* promoter in wild type in the dark to artificially control the growth of aerial hyphae (Fig 6B). Indeed, the thickness of aerial hyphae increased from 1.5 mm to 2.4 mm in the *fluG*^OE^ strain (Fig 6C and 6D). However, overexpression of the *fluG-*like gene did not cause a visible increase of aerial hyphal growth. In the dual confrontation assays, more DNA copies of the *fluG*^OE^ strain than wild type and the *fluG-like*^*OE*^ strain had been detected already after 5 days of incubation in the territory of *A*. *alternata*, *F*. *oxysporum*, and *F*. *fujikuroi* (Fig 6E and 6F), although for each of the phytopathogens the areas of their colony surface that were not covered by the *T*. *guizhouense* strains have no visible differences. After ten days, the *fluG*^OE^ strain not just occupied the territories of four phytopathogens, but also formed abundant green conidia on the colony surface of *A*. *aternata*, *F*. *oxysporum*, and *F*. *odoratissimum* in comparison to wild type and the *fluG-like*^*OE*^ strain (Fig 6E). Collectively, the *fluG*^OE^ strain exhibited enhanced antagonistic ability as compared to wild type and the *fluG-*like^OE^ strain. These results demonstrate that more abundant aerial hyphae can improve the antagonistic activity of *T*. *guizhouense*.

### Mutants with abundant aerial hyphae screened after UV-mutagenesis exhibit improved antagonistic activity

**Fig 7:**
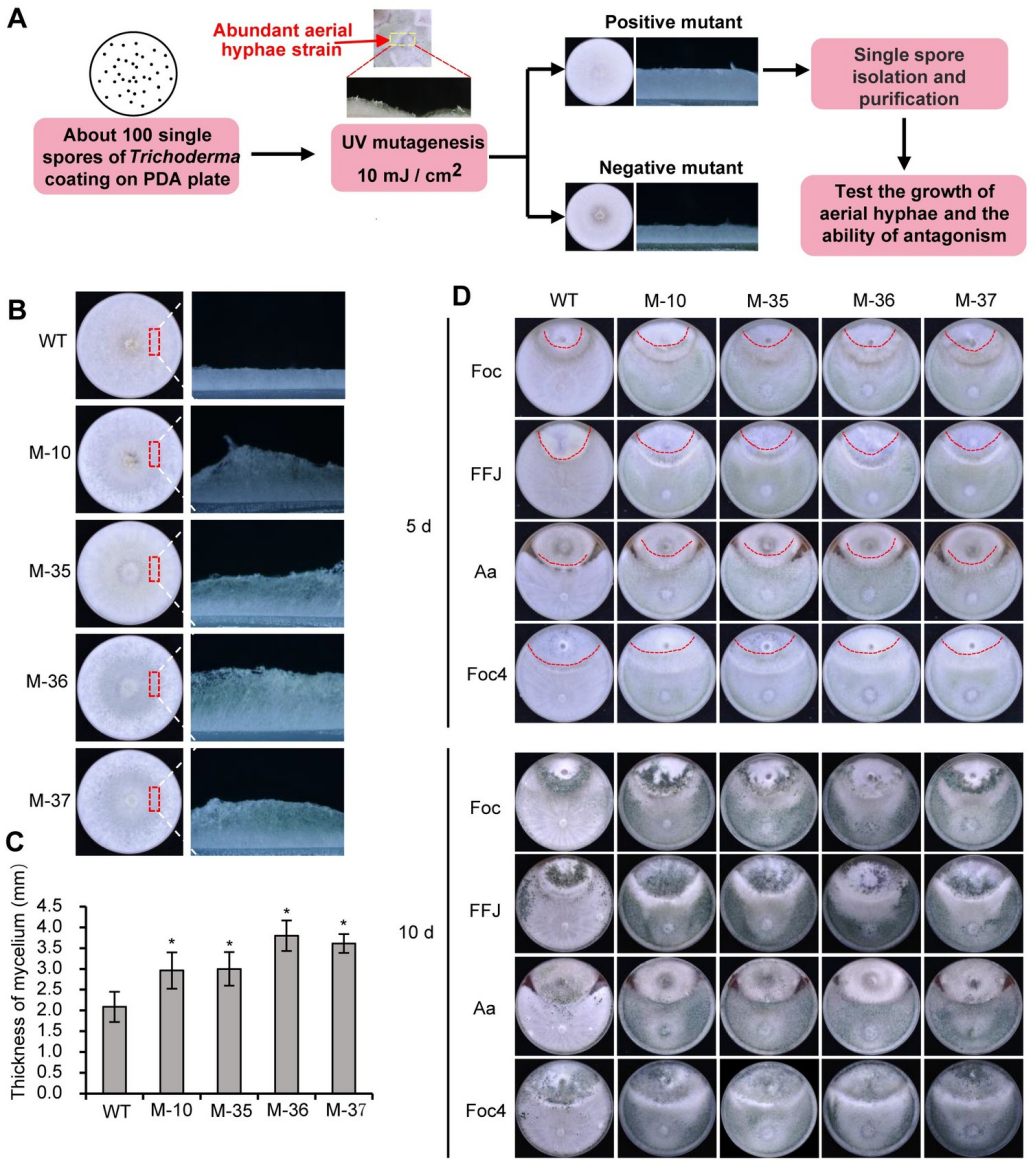
Screening for mutants with enhanced antagonistic activity. (A). Screening strategy for mutants with abundant aerial hyphae. Conidia were spread on a PDA plate (~100 spores/plate) and then treated with 10 mJ/cm^2^ UV light. All plates were incubated at 28°C in the dark for four days. The colonies with visible abundant aerial hyphae in comparison to the adjacent ones were isolated. Aerial hyphal growth and antagonistic ability of the selected mutants were further analyzed. (B). Phenotype of aerial hyphae of the mutants. All strains were incubated in the dark for 3 days. (C). Thickness measurement of aerial hyphae in wild type and nine mutants. Thickness of the aerial hyphae was determined as above. Error bar represents the standard deviation (SD) of five biological replicates. (D). Antagonistic ability analysis of the mutants. Mutants were confronted with the phytopathogenic fungi *A*. *alternata* (Aa), *F*. *oxysporum* (Foc), *F*. *fujikuroi* (FFJ) and *F*. *odoratissimum* (Foc4) for 5 or 10 days in the dark.

Given the importance of the aerial hyphae for the antagonistic activity of *T*. *guizhouense*, we speculated that there is a possibility to obtain mutants with improved antagonistic activity simply by isolating the colonies with visible abundant aerial hyphae after mutagenesis. Therefore, we performed UV-mutagenesis to screen for mutants with abundant aerial hyphae. After ultra-violet (UV) irradiation (10 mJ/cm^2^, survival rate of conidia: ~20%), PDA plates with conidia were incubated in the dark to avoid photoconidiation and the colonies with visible abundant aerial hyphae were screened (Fig 7A). Taken together, we identified 9 colonies from 260 plates, which formed much more aerial hyphae in comparison to adjacent ones (Figs 7B, 7C, S8A, and S8B). All of them produced more abundant aerial hyphae than the parent strain, and indeed 4 of them exhibited enhanced antagonistic activity against phytopathogenic fungi in the dual confrontation assays (Figs 7D and S8C). In the dual confrontation assays, the areas of colony surface of *A*. *alternata* and *F*. *odoratissimum* that were not covered by four mutants were smaller than those not covered by wild type after 5 days of incubation, and these mutants also produced more conidia than wild type when confronted with phytopathogens. This indicates from another approach that aerial hyphal growth in *T*. *guizhouense* is important for its antagonistic activity.

## Discussion

A number of *Trichoderma* species are antagonistic against phytopathogenic fungi and this antagonistic process is a combination of different mechanisms including mycoparasitism, competition for nutrients or space, and production of cell degrading enzymes as well as primary and secondary metabolites with antibiotic properties [23]. In this study, we show distinct antagonistic behaviors of *T*. *guizhouense* when confronted with different phytopathogenic fungi. It competed for growth space and probably also acted as a mycoparasite to feed on the mycelia of *A*. *alternata* as it overgrew the mycelial mat of *A*. *alternata*. By contrast, although *T*. *guizhouense* also overgrew *Fusarium* species, its mycelia penetrated the colonies of *Fusarium* species first and extended along the substrate surface, which is more like a competitive behavior for nutrients. The distinct antagonistic behaviors were likely due to the denser mycelial mat formed by *A*. *alternata*, so that the hyphae of *T*. *guizhouense* could not penetrate it easily. The results demonstrate the flexibility of the antagonistic attack of *Trichoderma* species, which probably depends on the mycelial characteristics of different phytopathogenic fungi.

**Fig 8:**
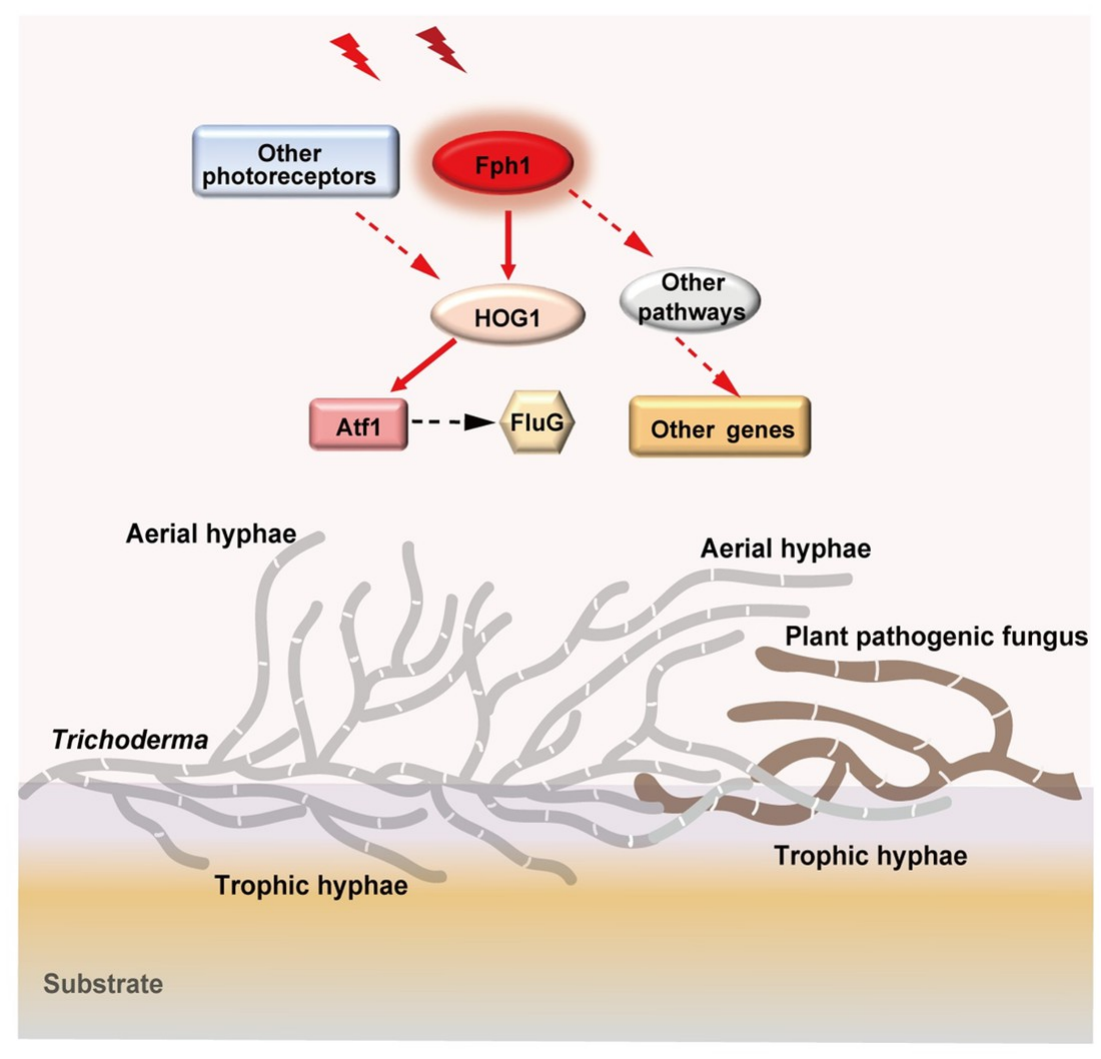
Model for the molecular mechanism of red and far-red light promoted aerial hyphal growth and the antagonistic ability of *T*. *guizhouense*. Red and far-red light signals are perceived by phytochrome FPH1, which further transmit the signals to the MAPK HOG pathway to cause phosphorylation of HOG1 and its translocation into the nucleus. The signals were finally transmitted to the bZIP transcription factor ATF1 that controls the expression of red and far-red light regulated genes including the *fluG* gene, resulting in more abundant aerial hyphae and the formation of conidiophores. Additionally, other light receptors or pathways also receive red and far-red light signals and trigger the differential expressions of some genes. Therefore, when *Trichoderma* and the phytopathogenic fungi live in the same habitat, aerial hyphae of *Trichoderma* grow faster in the present of red and far-red light signals from sunshine and more quickly penetrate or overgrow the colonies of the phytopathogenic fungi to feed on phytopathogenic fungi or compete for nutrients with them during antagonism. Consequently, under red and far-red light, growth of the phytopathogenic fungi is repressed more significantly, their territories are occupied more quickly, and more *Trichoderma* conidia for dispersal are formed.

Effects of environmental signals on the interspecies interactions or the mycoparasitic process remain largely unknown. One of the exciting findings in this study is the regulation of red and far-red light for the antagonistic ability of *T*. *guizhouense*, which becomes more aggressive under red and far-red light conditions during the antagonism against phytopathogenic fungi compared with the dark condition (Fig 8). Moreover, absence of FPH1 attenuated the antagonistic ability even in the dark, suggesting that FPH1 still functions in interspecific interactions even under both dark and light conditions. It was reported that the mycoparasitic ability of *T*. *atroviride* was better in darkness and upon yellow- or red-light exposure than in white light-dark cycle conditions when confronted with *F*. *oxysporum* [30]. Interestingly, when the blue light receptor BLR1 was absent, *T*. *atroviride* became more aggressive toward *B*. *cinerea* [31]. Therefore, light plays an important role for the antagonistic activity of *Trichoderma* spp. against phytopathogenic fungi. More abundant aerial hyphae formed under red and far-red light condition in *T*. *guizhouense* overgrew or penetrated the colonies of pathogens more quickly, resulting in the enhanced antagonistic ability of *T*. *guizhouense* (Fig 8). This was also verified by enhanced antagonistic ability of the *fluG*^*OE*^ strain and the mutants with abundant aerial hyphae.

Red and far-red light signaling and responses in *Trichoderma* spp. had not been studied in detail. While red light promotes the production of conidia in *A*. *nidulans* and *A*. *alternata*, it does not affect conidiation in *T*. *reesei* [7,10,14]. We show here that red and far-red light not only promote aerial hyphal growth but also activate conidiation in *T*. *guizhouense*, which is dependent on FPH1. It is interesting that red and far-red light have similar effects on these two processes, because red light promotes the conversion of phytochrome into the far-red-light form (Pfr), and far-red light converts it back to the red-light form (Pr), and thus the effects of red and far-red light are supposed to be different or even opposite. However, the similar effects of red and far-red light on conidiation were also observed in *B*. *bassiana* [15].

Our previous studies have deciphered the red-light signaling network in *A*. *nidulans* by transcriptomic profiling, in which the expression of light-regulated genes is mainly orchestrated by phytochrome FphA and the MAPK HOG (SAK) pathway [12,13]. Nevertheless, light induction of a small proportion of genes is not governed by FphA, suggesting alternative mechanisms for red and far-red light sensing. By contrast, in the smut fungus *Ustilago maydis*, after 60 min illumination, 77 and 114 DEGs identified in red and far-red light, respectively, were all strictly regulated by phytochrome, as no DEGs were identified in the phytochrome deletion strain [32]. The results here demonstrated in *T*. *guizhouense* both red and far-red plug into HOG pathway via FPH1, whereas deletion of *fph1* reduced the phosphorylation level of HOG1 but not disrupted it completely. In agreement with this, on the transcriptomic level, totals of 66.0% (268/406) and 55.1% (343/623) of DEGs identified in wild type under red and far-red light conditions, respectively, were regulated by FPH1, and in the *Δfph1* strain a large array of genes were still differentially expressed. Hence, *T*. *guizhouense* may employ alternative mechanisms to transmit red or far-red light signals.

It appears that *Trichoderma* species are more sensitive to far-red light, as more aerial hyphae in both *T*. *guizhouense* and *T*. *virens* were formed under far-red light condition than under red light condition. Consistently, far-red light controls more genes than red light in *T*. *guizhouense*. Similarly, as abovementioned, more DEGs were identified in far-red light than in red light in *U*. *maydis* [32]. However, in *A*. *nidulans*, red light governs the expression of more genes, where 1005 genes were differentially expressed after 15 min illumination of red light in comparison to 450 upon far-red light illumination [13]. Additionally, although corresponding transcriptome analyses still lack, *A*. *alternata* exhibits stronger phenotypic responses in far-red light than in red light [14].

MAPK HOG (SAK) pathway is a hub of different environmental cues, which is involved in the signaling of osmotic and oxidative stresses, heat shock and light [2,12,29,33,34]. We show here that 67.9% of red-light regulated and 74.6% of far-red light regulated genes are governed by HOG1 in *T*. *guizhouense*, respectively. It is surprising that much more DEGs were identified in red (1124 DEGs) and far-red (1286 DEGs) light in the *Δhog1* strain than in the wild type strain. By contrast, in *A*. *nidulans* only 33 genes were differentially expressed upon exposure of red light in the *ΔhogA* strain. It suggests the alternative pathway for red and far-red light signaling in *T*. *guizhouense*. Moreover, our previous study demonstrated that the white collar-1 ortholog BLR1 utilizes HOG pathway to transmit bule light signal [35]. Hence, how the different environmental signals converge and then diverge at HOG1 is worth further investigation.

Importantly, it is very easy to pick out the mutants with visible abundant aerial hyphae from hundreds of colonies after UV mutagenesis. This simple screening method can be applied definitely to other *Trichoderma* species to develop the mutants with better biocontrol performances, which will facilitate the application of biocontrol agents (BCAs) in agriculture.

## Materials and methods

### Strains and culture conditions

*T*. *guizhouense* NJAU4742 and four phytopathogenic fungi *Fusarium odoratissimum* (syn: *Fusarium oxysporum* f. sp. *cubense* race 4), *Fusarium fujikuroi*, *Fusarium oxysporum* CIPP1012 and *Alternaria alternata* were used in this study. All strains were cultured with PDA or PDB media (BD Difco, USA) at 28°C under dark or light conditions in light boxes previously designed [12] and the light intensity was 1.7 μmol photons/(m^2^×s).

The construction of the *Δhog1* strain and the strain expressing HOG1-EGFP fusion protein was described in a previous study [35]. To delete the *fph1* gene in *T*. *guizhouense*, the hygromycin B phosphotransferase (*hph*) gene cassette was fused with *fph1* upstream (1989 bp) and downstream (2660 bp) fragments in pUC19 with Clonexpress MultiS One Step Cloning Kit (Vazyme, China). Transformation was performed as previously described [26]. Positive transformants were confirmed by diagnostic PCR and Southern blot (S1A Fig). The mutant was complemented ectopically with the plasmid pST2 harboring the geneticin resistance (*neo*) gene cassette and the *fph1* gene, yielding the strain *fph1*^C^. To overexpress the *fluG* and the *fluG*-like genes, *T*. *reesei cdna1* promoter, ORFs and their terminator regions of both genes and the *hph* gene were assembled in pUC19, yielding the plasmids pST7 and pST8, respectively. Positive transformants (*fluG*^OE^ and *fluG-*like^OE^) were confirmed by PCR and quantitative real-time PCR. Strains and primers are listed in S1 and S2 Tables, respectively.

### Dual confrontation assays

Mycelial plugs (6 mm in diameter) of each phytopathogenic fungus were placed separately on PDA plates (9 cm in diameter), 2 cm away from the edge, and incubated in the dark for two days at 28°C. Subsequently, mycelial plugs of the *T*. *guizhouense* strains were placed separately on the opposite side of the colonies of phytopathogenic fungi. The plates were then incubated at 28°C under different light conditions for 5 or 10 days.

To assess the DNA copies of the wild type and the *Δfph1* strains in the territories of the host fungi in confrontation assays, mixed mycelia composed of both overlaid *T*. *guizhouense* and the host fungi were sampled for DNA extraction using the Fungal DNA Kit (Omega, USA). The strain-specific primers P37/P38 and methods were performed as previously described [27]. The colony surface areas of four phytopathogens were analyzed using the software ImageJ.

### RNA extraction and RT-qPCR

Wild type and mutants were cultivated on PDA plate covered with cellophane at 28°C in the dark, red and far-red light for three days. Collected mycelia were immediately frozen in liquid nitrogen for RNA isolation using SteadyPure Plant RNA Extraction Kit (Accurate Biotechnology, Hunan, China). cDNA was synthesized with HiScript III 1st Strand cDNA Synthesis Kit (+gDNA wiper) (Vazyme). qPCR was performed on a qTOWER 2.2 system (Jena, Germany) and the relative expression levels of genes were calculated with the 2^-ΔCt^ method using *tef1* as the housekeeping gene.

### Transcriptome sequencing and analyses

Wild type, *Δfph1*, and *Δhog1* were cultivated on PDA plates covered with cellophane at 28°C in the dark for 24 h and then exposed to red light and far-red light or kept in the dark for 45 min before RNA isolation. Samples of total mRNA were subjected to RNA sequencing that was performed by Gene Denovo Biotechnology Co., Ltd. (Guangzhou, China). The sequence reads were mapped onto the reference genome of *T*. *guizhouense* using HISAT 2.2.4 with the default parameters. For each transcript, a fragment per kilobase of transcript per million mapped reads value was calculated to quantify its expression abundance and variations, using the StringTie v1.3.1. DEGs were identified using DEGseq2 with thresholds of |log2(fold change)| ≥1 and FDR <0.05. GO enrichment and KEGG pathway enrichment analyses were carried out on Omicshare platform (https://www.omicshare.com/tools/). The sequencing data are available in the NCBI database Sequencing Read Archive under the accession number PRJNA954062.

### Microscopy

To analyze the subcellular localization of HOG1, fresh conidia of the strain expressing HOG1-EGFP were inoculated on coverslips with 400 μl PDB medium. After 14 h of incubation in the dark at 28°C, samples were exposed to red or far-red light for 3 min or kept in the dark. Samples were then fixed immediately with 4% formaldehyde for 10 min and washed twice with 1 × PBS (pH 7.4). Nuclei were stained with Hoechst (no. C0031, Solarbio) before microscopy. EGFP signals were observed with a confocal laser scanning microscope (Leica TCS SP8, Germany).

### Immunofluorescence

Immunofluorescence was performed as previously described [35]. Fresh conidia (1 × 10^5^ spores/ml) of the wild type and the *Δfph1* strains were inoculated on coverslips with 400 μl PDB medium and cultured overnight in the dark at 28°C. The samples were then exposed to red or far-red light for 5 min or kept in the dark before fixation. Antiphospho-p38 MAP kinase (Thr180/ Tyr182) antibodies (no. 9211, 1:400 dilution; Cell Signaling Technology) against phosphorylated HOG1, and secondary antibody Cy3-conjugated anti-rabbit IgG (1:100 dilution, Sangon Biotech, Shanghai, China) were used for immunofluorescence staining. Nuclei were stained with Hoechst (no. C0031, Solarbio).

### Western blot analysis

Fresh spores (1 × 10^5^ spores/ml) were cultured in 50 ml PDB (BD Difco, USA) medium at 28°C in the dark under agitation at 170 rpm for 24 h. Mycelia were harvested and frozen in liquid nitrogen immediately after 5-, 10- and 20-min of red or far-red light treatments, respectively. Protein extraction and Western blot were performed as described previously [12]. Antibodies used here were antiphospho-p38 MAP kinase (Thr180/ Tyr182) antibodies (no. 9211, 1:1000 dilution; Cell Signaling Technology) against phosphorylated HOG1, anti-p38 MAP kinase antibody (no. 9212, 1:1000 dilution; Cell Signaling Technology) against HOG1, and anti-rabbit HRP-labeled secondary antibody (no. A0208, Beyotime, China).

## Supporting information

S1 Fig Construction of the *Δfph1-*mutant strain. (A). Domain prediction of *A*. *nidulans* FphA and *T*. *guizhouense* FPH1. Sequence identity: 52%, e-value: 2e-46. (B). Southern blot analysis of the positive *Δfph1-*mutant strain.
(PDF)

S2 Fig Phenotype of aerial hyphae of the *Δfph1* and the *fph1*^*C*^ strains. (A). Aerial hyphae growth analysis of wild type, *Δfph1*, and *fph1*^*C*^ strains. All strains were cultivated on PDA plates under dark or light conditions. The hyphae were observed under a stereoscope. (B). Thickness measurement of aerial hyphae of the wild type, *Δfph1*, and *fph1*^*C*^ strains. Error bars represent the SD of five biological replicates. Statistically significant difference was evaluated by Student’s *t*-test: * *p* < 0.05; ** *p* < 0.01; *** *p* < 0.001; **** *p* < 0.0001; n.s. means no significant difference.
(PDF)

S3 Fig Conidiation of wild type, *Δfph1* and *Δhog1* strains in response to red and far-red light. (A). Phenotypes of wild type, *Δfph1* and *Δhog1* strains in red and far-red light. All strains were cultured on 9 cm PDA plates at 28°C under different light conditions for five days. (B). Quantification of conidia of each strain. Mean values for the three samples biological replicates were displayed. Bars present mean values ± SD. Statistically significant difference is evaluated by Student’s *t*-test: * *p* < 0.05; ** *p* < 0.01; *** *p* < 0.001; **** *p* < 0.0001.
(PDF)

S4 Fig Validation of transcriptome data using RT-qPCR. (A). Expression level of DEGs dependent on HOG1. The mean values were calculated from three independent biological replicates and the error bars represent the standard deviation (SD). (B). Expression level of DEGs dependent on both FPH1 and HOG1. (C). Expression level of DEGs dependent on neither FPH1 nor HOG1. (D). DEGs repressed by red and far-red light.
(PDF)

S5 Fig Domain arrangements of *A*. *nidualans* FluG and its homologs in *T*. *guizhouense*. A blast search against the genome with the protein sequence of *A*. *nidulans* FluG as query was performed. Two putative proteins OPB45185 and OPB45752 with high similarity to *A*. *nidulans* FluG (Sequence identity: OPB45185, 30%, e-value: 3e-61; OPB45752, 31%, e-value: 2e-54) were identified and referred to as *fluG* and *fluG-like*, respectively.
(PDF)

S6 Fig Antagonistic activity of the *Δhog1* and *Δatf1* mutants under red and far-red light conditions. Wild type, *Δhog1* and *Δatf1* strains were confronted with the phytopathogenic fungi at 28°C for 5 days under different light conditions.
(PDF)

S7 Fig Two replicates of Western blot for the detection of HOG1 phosphorylation detection under different light conditions. (PDF)

S8 Fig Phenotype of aerial hyphae and antagonistic activity of five mutants screened via UV-mutagenesis. (A). Phenotype of aerial hyphae of the mutants. (B). Thickness measurement of aerial hyphae in wild type and five mutants. Error bar represents the standard deviation (SD) of five biological replicates. (C). Antagonistic ability analysis of the mutants. Mutants were confronted with the phytopathogenic fungi *A*. *alternata* (Aa), *F*. *oxysporum* (Foc), *F*. *fujikuroi* (FFJ) and *F*. *odoratissimum* (Foc4) at 28°C for 5 or 10 days in the dark.
(PDF)

S1 Table Strains used in this study. (XLSX)

S2 Table Primers used in this study. (XLSX)

S3 Table Top 10 enriched Go terms of DEGs identified in wild type. (XLSX)

## References

[1] Herrera-Estrella A, Horwitz BA. Looking through the eyes of fungi: molecular genetics of photoreception. Molecular Microbiology. 2007 Apr;64(1):5–15. doi: 10.1111/j.1365-2958.2007.05632.x 17376067

[2] Rodriguez-Romero J, Hedtke M, Kastner C, Müller S, Fischer R. Fungi, Hidden in Soil or Up in the Air: Light Makes a Difference. Annu Rev Microbiol. 2010 Oct 13;64(1):585–610. doi: 10.1146/annurev.micro.112408.134000 20533875

[3] Yu Z, Fischer R. Light sensing and responses in fungi. Nature Reviews Microbiology. 2019 Jan 1;17(1):25–36. doi: 10.1038/s41579-018-0109-x 30377305

[4] Corrochano LM. Light in the Fungal World: From Photoreception to Gene Transcription and Beyond. Annu Rev Genet. 2019 Dec 3;53(1):149–70. doi: 10.1146/annurev-genet-120417-031415 31451036

[5] Tisch D, Schmoll M. Light regulation of metabolic pathways in fungi. Appl Microbiol Biotechnol. 2010 Feb;85(5):1259–77. doi: 10.1007/s00253-009-2320-1 19915832 PMC2807966

[6] Bayram ÖS, Bayram Ö. An Anatomy of Fungal Eye: Fungal Photoreceptors and Signalling Mechanisms. Journal of Fungi. 2023 May 19;9(5):591. doi: 10.3390/jof9050591 37233302 PMC10219052

[7] Schmoll M, Esquivel-Naranjo EU, Herrera-Estrella A. *Trichoderma* in the light of day—Physiology and development. Fungal Genetics and Biology. 2010;8. 10.1016/j.fgb.2010.04.010 PMC2954361 20466064

[8] Schumacher J. How light affects the life of *Botrytis*. Fungal Genetics and Biology. 2017 Sep; 106:26–41. 28648816 10.1016/j.fgb.2017.06.002

[9] Fuller KK, Loros JJ, Dunlap JC. Fungal photobiology: visible light as a signal for stress, space and time. Curr Genet. 2015 Aug;61(3):275–88. doi: 10.1007/s00294-014-0451-0 25323429 PMC4401583

[10] Blumenstein A, Vienken K, Tasler R, Purschwitz J, Veith D, Frankenberg-Dinkel N, et al. The *Aspergillus nidulans* Phytochrome FphA Represses Sexual Development in Red Light. Current Biology. 2005 Oct;15(20):1833–8. 16243030 10.1016/j.cub.2005.08.061

[11] Purschwitz J, Müller S, Kastner C, Schöser M, Haas H, Espeso EA, et al. Functional and Physical Interaction of Blue- and Red-Light Sensors in *Aspergillus nidulans*. Current Biology. 2008 Feb;18(4):255–9. 18291652 10.1016/j.cub.2008.01.061

[12] Yu Z, Armant O, Fischer R. Fungi use the SakA (HogA) pathway for phytochrome-dependent light signalling. Nature Microbiology. 2016 May;1(5):16019. doi: 10.1038/nmicrobiol.2016.19 27572639

[13] Yu Z, Streng C, Seibeld RF, Igbalajobi OA, Leister K, Ingelfinger J, et al. Genome-wide analyses of light-regulated genes in *Aspergillus nidulans* reveal a complex interplay between different photoreceptors and novel photoreceptor functions. PLoS Genet. 2021 Oct 22;17(10):e1009845. 34679095 10.1371/journal.pgen.1009845 PMC8535378

[14] Igbalajobi O, Yu Z, Fischer R. Red- and Blue-Light Sensing in the Plant Pathogen *Alternaria alternata* Depends on Phytochrome and the White-Collar Protein LreA. mBio. 2019 Apr 9;10(2):e00371–19. 30967462 10.1128/mBio.00371-19 PMC6456751

[15] Qiu L, Wang JJ, Chu ZJ, Ying SH, Feng MG. Phytochrome controls conidiation in response to red/far-red light and daylight length and regulates multistress tolerance in *Beauveria bassiana*: Functional diversity of a fungal phytochrome. Environ Microbiol. 2014 Jul;16(7):2316–28. 24725588 10.1111/1462-2920.12486

[16] Yu JH, Mah JH, Seo JA. Growth and Developmental Control in the Model and Pathogenic *Aspergilli*. Eukaryotic Cell. 2006 Oct;5(10):1577–84. 17030989 10.1128/EC.00193-06 PMC1595332

[17] Heitman Howlett, Crous Stukenbrock, James Gow, editors. Cell Biology of Hyphal Growth. In: The Fungal Kingdom. American Society of Microbiology; 2017. pp. 231–65.

[18] Meyer V, Basenko EY, Benz JP, Braus GH, Caddick MX, Csukai M, et al. Growing a circular economy with fungal biotechnology: a white paper. Fungal Biol Biotechnol. 2020 Dec;7(1):5. doi: 10.1186/s40694-020-00095-z 32280481 PMC7140391

[19] Park HS, Yu JH. Genetic control of asexual sporulation in filamentous fungi. Current Opinion in Microbiology. 2012 Dec;15(6):669–77. doi: 10.1016/j.mib.2012.09.006 23092920

[20] Etxebeste O., Garzia A., Espeso E. A., and Ugalde U., 2010 *Aspergillus nidulans* asexual development: making the most of cellular modules. Trends Microbiol. 18: 569–576. 21035346 10.1016/j.tim.2010.09.007

[21] Ruger-Herreros C, Rodríguez-Romero J, Fernández-Barranco R, Olmedo M, Fischer R, Corrochano LM, et al. Regulation of Conidiation by Light in *Aspergillus nidulans*. Genetics. 2011 Aug;188(4):809–22. 21624998 10.1534/genetics.111.130096 PMC3176103

[22] Adams TH, Hide WA, Yager LN, Lee BN. Isolation of a Gene Required for Programmed Initiation of Development by *Aspergillus nidulans*. Molecular and Cellular Biology. 1992 Sep 1;12(9):3827–33. 1508186 10.1128/mcb.12.9.3827-3833.1992 PMC360252

[23] Druzhinina IS, Seidl-Seiboth V, Herrera-Estrella A, Horwitz BA, Kenerley CM, Monte E, et al. *Trichoderma*: the genomics of opportunistic success. Nat Rev Microbiol. 2011 Oct;9(10):749–59. 21921934 10.1038/nrmicro2637

[24] Woo SL, Hermosa R, Lorito M, Monte E. *Trichoderma*: a multipurpose, plant-beneficial microorganism for eco-sustainable agriculture. Nat Rev Microbiol. 2023 May;21(5):312–26. 36414835 10.1038/s41579-022-00819-5

[25] Wrzosek M, Ruszkiewicz-Michalska M, Sikora K, Damszel M, Sierota Z. The plasticity of fungal interactions. Mycol Progress. 2017 Feb;16(2):101–8.

[26] Zhang J, Miao Y, Rahimi MJ, Zhu H, Steindorff A, Schiessler S, et al. Guttation capsules containing hydrogen peroxide: an evolutionarily conserved NADPH oxidase gains a role in wars between related fungi. Environ Microbiol. 2019 Aug;21(8):2644–58. doi: 10.1111/1462-2920.14575 30815928 PMC6850483

[27] Zhu H, Zhang J, Gao Q, Pang G, Sun T, Li R, et al. A new atypical short-chain dehydrogenase is required for interfungal combat and conidiation in *Trichoderma guizhouense*. Environmental Microbiology. 2021 Oct;23(10):5784–801. doi: 10.1111/1462-2920.15493 33788384

[28] Pang G, Sun T, Yu Z, Yuan T, Liu W, Zhu H, et al. Azaphilones biosynthesis complements the defence mechanism of *Trichoderma guizhouense* against oxidative stress. Environ Microbiol. 2020 Nov;22(11):4808–4824. 32985773 10.1111/1462-2920.15246

[29] Lara-Rojas F, Sánchez O, Kawasaki L, Aguirre J. *Aspergillus nidulans* transcription factor AtfA interacts with the MAPK SakA to regulate general stress responses, development and spore functions: The SakA-AtfA pathway in stress and spore function. Molecular Microbiology. 2011 Apr;80(2):436–54. 21320182 10.1111/j.1365-2958.2011.07581.x PMC3108070

[30] Moreno-Ruiz D, Fuchs A, Missbach K, Schuhmacher R, Zeilinger S. Influence of Different Light Regimes on the Mycoparasitic Activity and 6-Pentyl-α-pyrone Biosynthesis in Two Strains of *Trichoderma atroviride*. Pathogens. 2020 Oct 21;9(10):860. 33096850 10.3390/pathogens9100860 PMC7589932

[31] Henríquez-Urrutia M, Spanner R, Olivares-Yánez C, Seguel-Avello A, Pérez-Lara R, Guillén-Alonso H, et al. Circadian oscillations in *Trichoderma atroviride* and the role of core clock components in secondary metabolism, development, and mycoparasitism against the phytopathogen *Botrytis cinerea*. eLife. 2022 Aug 11;11:e71358. 35950750 10.7554/eLife.71358 PMC9427114

[32] Brych A, Haas FB, Parzefall K, Panzer S, Schermuly J, Altmüller J, et al. Coregulation of gene expression by White collar 1 and phytochrome in *Ustilago maydis*. Fungal Genetics and Biology. 2021 Jul; 152:103570. 34004340 10.1016/j.fgb.2021.103570

[33] Esquivel-Naranjo EU, García-Esquivel M, Medina-Castellanos E, Correa-Pérez VA, Parra-Arriaga JL, Landeros-Jaime F, et al. A *Trichoderma atroviride* stress-activated MAPK pathway integrates stress and light signals: *Trichoderma* integrated stress and light signalling. Molecular Microbiology. 2016 Jun;100(5):860–76. 26878111 10.1111/mmi.13355

[34] Konte T, Terpitz U, Plemenitaš A. Reconstruction of the High-Osmolarity Glycerol (HOG) Signaling Pathway from the Halophilic Fungus *Wallemia ichthyophaga* in *Saccharomyces cerevisiae*. Frontiers in Microbiology. 2016 Jun 13; 7:901. 27379041 10.3389/fmicb.2016.00901 PMC4904012

[35] Li Y, Sun T, Guo D, Gao J, Zhang J, Cai F, et al. Comprehensive analysis of the regulatory network of blue-light-regulated conidiation and hydrophobin production in *Trichoderma guizhouense*. Environ Microbiol. 2021 Oct; 23(10): 6241–6256. 34472181 10.1111/1462-2920.15748

