# Supplementary material for: Red and far-red light improve the antagonistic ability of Trichoderma guizhouense against phytopathogenic fungi by promoting phytochrome-dependent aerial hyphal growth
Source: PLoS Genet. 2024 May 20;20(5):e1011282. doi: 10.1371/journal.pgen.1011282 (PMC11142658; doi:10.1371/journal.pgen.1011282)
Supplement: S1 Fig — (A). Domain prediction of A. nidulans FphA and T. guizhouense FPH1. Sequence identity: 52%, e-value: 2e-46. (B). Southern blot analysis of the positive Δfph1-mutant strain. (PDF) [file pgen.1011282.s001.pdf]

S1 Fig

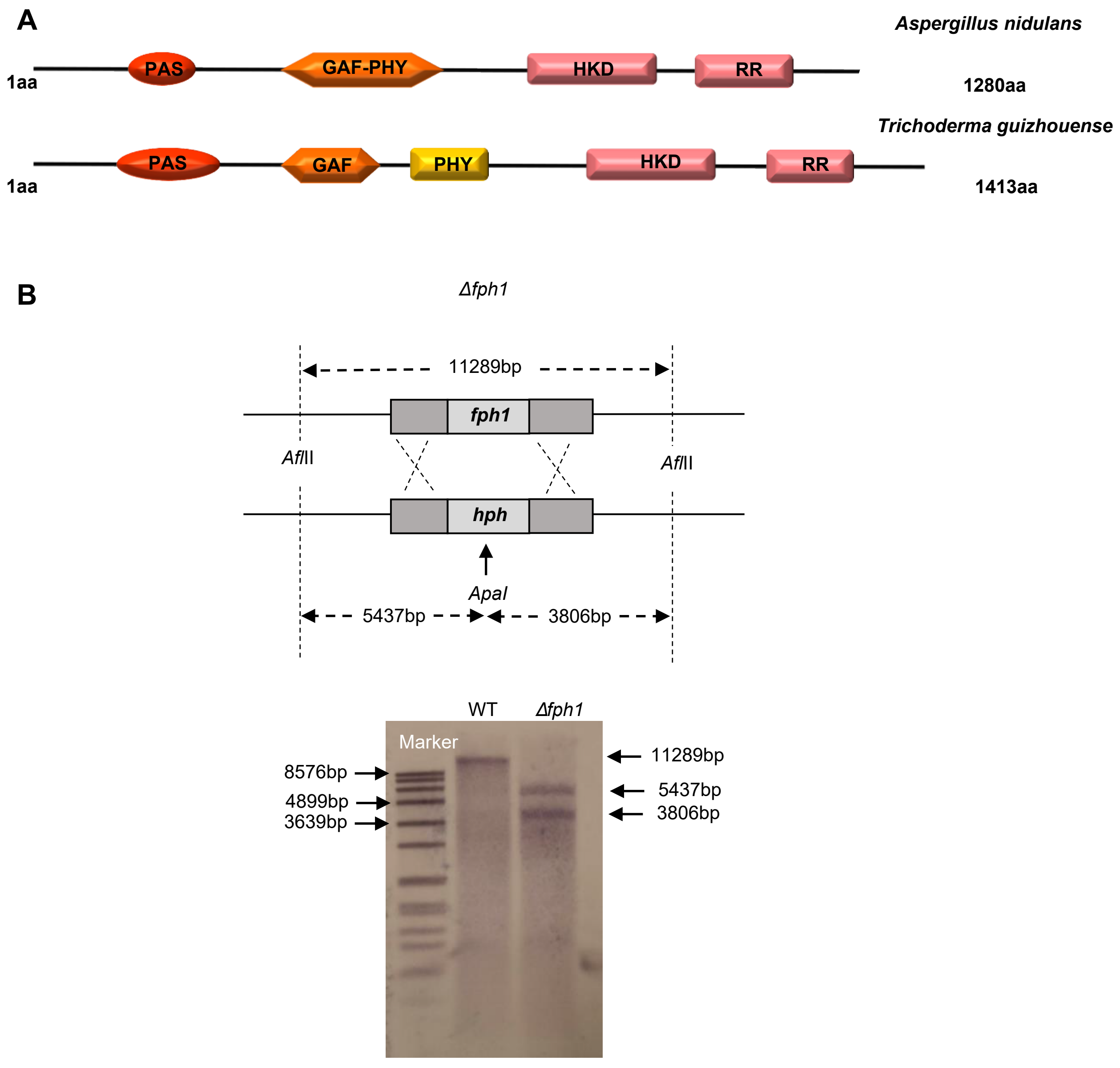

S1 Fig. Construction of the *Δfph1*-mutant strain.
(A). Domain prediction of *A. nidulans* FphA and *T. guizhouense* FPH1. Sequence identity: 52%, e-value: 2e-46. (B). Southern blot analysis of the positive *Δfph1*-mutant strain.
