# Supplementary material for: Red and far-red light improve the antagonistic ability of Trichoderma guizhouense against phytopathogenic fungi by promoting phytochrome-dependent aerial hyphal growth
Source: PLoS Genet. 2024 May 20;20(5):e1011282. doi: 10.1371/journal.pgen.1011282 (PMC11142658; doi:10.1371/journal.pgen.1011282)
Supplement: S2 Fig — (A). Aerial hyphae growth analysis of wild type, Δfph1, and fph1C strains. All strains were cultivated on PDA plates under dark or light conditions. The hyphae were observed under a stereoscope. (B). Thickness measurement of aerial hyphae of the wild type, Δfph1, and fph1C strains. Error bars represent the SD of five biological replicates. Statistically significant difference was evaluated by Student’s t-test: * p < 0.05; ** p < 0.01; *** p < 0.001; **** p < 0.0001; n.s. means no significant difference. (PDF) [file pgen.1011282.s002.pdf]

S2 Fig

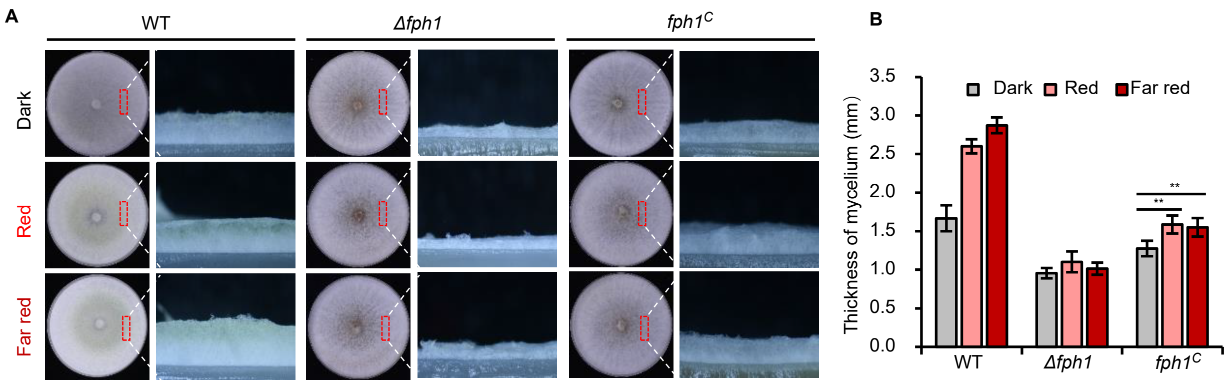

S2 Fig. Phenotype of aerial hyphae of the *Δfph1* and the fph1C strains.

(A). Aerial hyphae growth analysis of wild type, *Δfph1*, and *fph1*^{C} strains. All strains were cultivated on PDA plates under dark or light conditions. The hyphae were observed under a stereoscope. (B). Thickness measurement of aerial hyphae of the wild type, *Δfph1*, and *fph1*^{C} strains. Error bars represent the SD of five bio-logical replicates. Statistically significant difference was evaluated by Student's t-test: * $p < 0.05$; ** $p < 0.01$; *** $p < 0.001$; **** $p < 0.0001$; n.s. means no signif-icant difference.
