# Supplementary material for: Red and far-red light improve the antagonistic ability of Trichoderma guizhouense against phytopathogenic fungi by promoting phytochrome-dependent aerial hyphal growth
Source: PLoS Genet. 2024 May 20;20(5):e1011282. doi: 10.1371/journal.pgen.1011282 (PMC11142658; doi:10.1371/journal.pgen.1011282)
Supplement: S3 Fig — (A). Phenotypes of wild type, Δfph1 and Δhog1 strains in red and far-red light. All strains were cultured on 9 cm PDA plates at 28°C under different light conditions for five days. (B). Quantification of conidia of each strain. Mean values for the three samples biological replicates were displayed. Bars present mean values ± SD. Statistically significant difference is evaluated by Student’s t-test: * p < 0.05; ** p < 0.01; *** p < 0.001; **** p < 0.0001. (PDF) [file pgen.1011282.s003.pdf]

S3 Fig

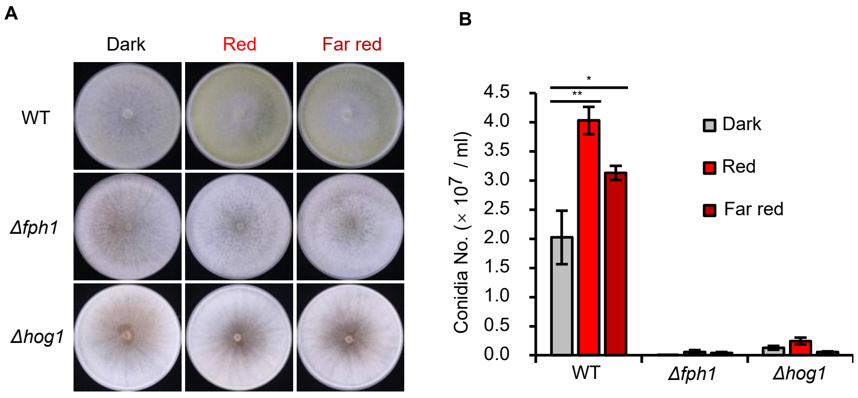

S3 Fig. Conidiation of wild type, *Δfph1* and *Δhog1* strains in response to red and far-red light. (A) Phenotypes of wild type, *Δfph1* and *Δhog1* strains in red and far-red light. All strains were cultured on 9 cm PDA plates at 28 °C under different light conditions for five days. (B) Quantification of conidia of each strain. Mean values for the three samples biological replicates were displayed. Bars present mean values ± SD. Statistically significant difference is evaluated by Student's t-test: * $p < 0.05$; ** $p < 0.01$; *** $p < 0.001$; **** $p < 0.0001$.
