# Supplementary material for: Red and far-red light improve the antagonistic ability of Trichoderma guizhouense against phytopathogenic fungi by promoting phytochrome-dependent aerial hyphal growth
Source: PLoS Genet. 2024 May 20;20(5):e1011282. doi: 10.1371/journal.pgen.1011282 (PMC11142658; doi:10.1371/journal.pgen.1011282)
Supplement: S4 Fig — (A). Expression level of DEGs dependent on HOG1. The mean values were calculated from three independent biological replicates and the error bars represent the standard deviation (SD). (B). Expression level of DEGs dependent on both FPH1 and HOG1. (C). Expression level of DEGs dependent on neither FPH1 nor HOG1. (D). DEGs repressed by red and far-red light. (PDF) [file pgen.1011282.s004.pdf]

S4 Fig

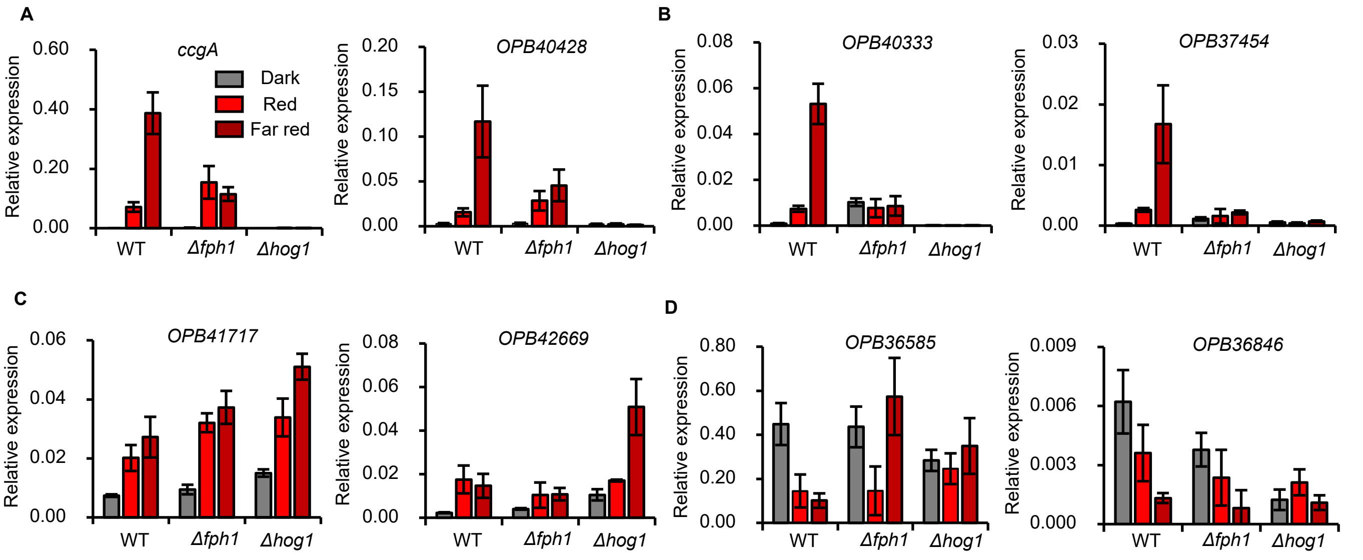

S4 Fig. Validation of transcriptome data using RT-qPCR. (A) Expression level of DEGs dependent on HOG1. The mean values were cal-culated from three independent biological replicates and the error bars represent the standard deviation (SD). (B) Expression level of DEGs dependent on both FPH1 and HOG1. (C) Expression level of DEGs dependent on neither FPH1 nor HOG1. (D)Expression level of DEGs repressed by red and far-red light.
