# Supplementary material for: Red and far-red light improve the antagonistic ability of Trichoderma guizhouense against phytopathogenic fungi by promoting phytochrome-dependent aerial hyphal growth
Source: PLoS Genet. 2024 May 20;20(5):e1011282. doi: 10.1371/journal.pgen.1011282 (PMC11142658; doi:10.1371/journal.pgen.1011282)
Supplement: S5 Fig — A blast search against the genome with the protein sequence of A. nidulans FluG as query was performed. Two putative proteins OPB45185 and OPB45752 with high similarity to A. nidulans FluG (Sequence identity: OPB45185, 30%, e-value: 3e-61; OPB45752, 31%, e-value: 2e-54) were identified and referred to as fluG and fluG-like, respectively. (PDF) [file pgen.1011282.s005.pdf]

S5 Fig

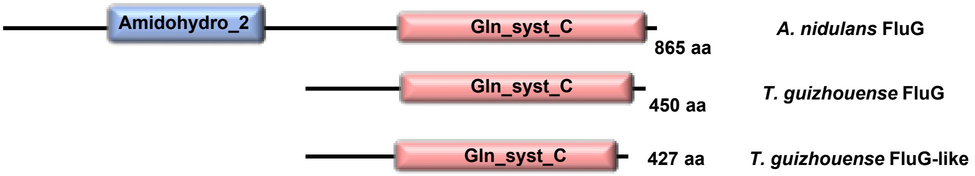

S5 Fig. Domain arrangements of *A. nidualans* FluG and its homologs in *T. guizhouense.* Blast search against the genome with the protein sequence of *A. nidulans* FluG as query was performed. Two putative proteins OPB45185 and OPB45752 with high similarity to *A. nidulans* FluG (Sequence identity: OPB45185, 30%, e-value: 3e-61; OPB45752, 31%, e-value: 2e-54) were identified and referred to as *fluG* and *fluG-like*, respectively.
