# Supplementary material for: Red and far-red light improve the antagonistic ability of Trichoderma guizhouense against phytopathogenic fungi by promoting phytochrome-dependent aerial hyphal growth
Source: PLoS Genet. 2024 May 20;20(5):e1011282. doi: 10.1371/journal.pgen.1011282 (PMC11142658; doi:10.1371/journal.pgen.1011282)
Supplement: S6 Fig — Wild type, Δhog1 and Δatf1 strains were confronted with the phytopathogenic fungi at 28°C for 5 days under different light conditions. (PDF) [file pgen.1011282.s006.pdf]

S6 Fig

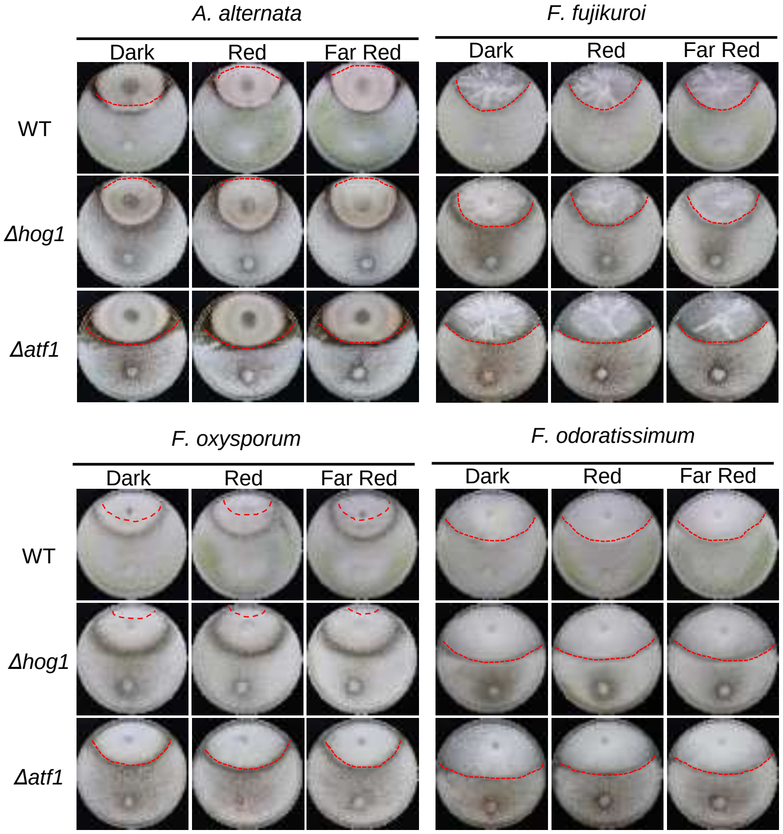

S6 Fig. Antagonistic activity of the *Δhog1* and *Δatf1* mutants under red and far-red light conditions.
Wild type, *Δhog1* and *Δatf1* strains were confronted with the phytopathogenic fungi at 28 °C for 5 days under different light conditions.
