# Supplementary material for: Red and far-red light improve the antagonistic ability of Trichoderma guizhouense against phytopathogenic fungi by promoting phytochrome-dependent aerial hyphal growth
Source: PLoS Genet. 2024 May 20;20(5):e1011282. doi: 10.1371/journal.pgen.1011282 (PMC11142658; doi:10.1371/journal.pgen.1011282)
Supplement: S7 Fig — (PDF) [file pgen.1011282.s007.pdf]

S7 Fig

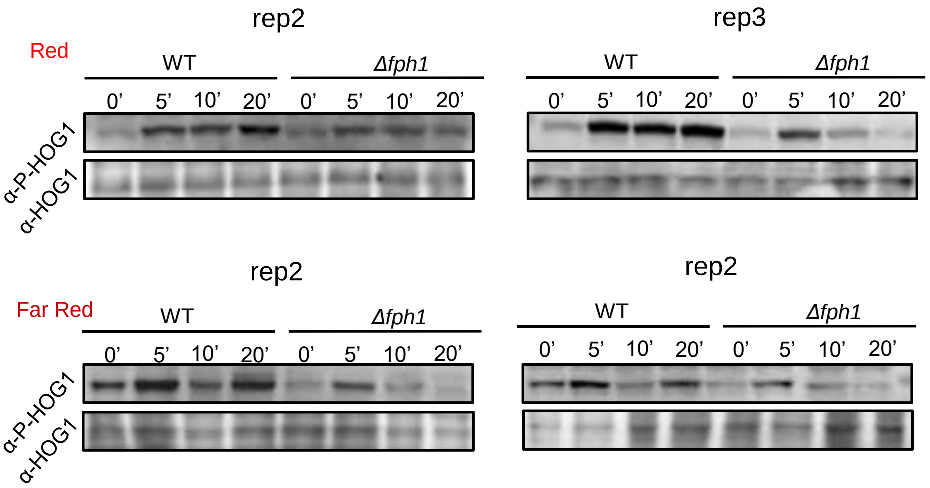

S7 Fig. Two replicates of western blot for the detection of HOG1 phos-phorylation detection under different light conditions.
