# Supplementary material for: Red and far-red light improve the antagonistic ability of Trichoderma guizhouense against phytopathogenic fungi by promoting phytochrome-dependent aerial hyphal growth
Source: PLoS Genet. 2024 May 20;20(5):e1011282. doi: 10.1371/journal.pgen.1011282 (PMC11142658; doi:10.1371/journal.pgen.1011282)
Supplement: S8 Fig — (A). Phenotype of aerial hyphae of the mutants. (B). Thickness measurement of aerial hyphae in wild type and five mutants. Error bar represents the standard deviation (SD) of five biological replicates. (C). Antagonistic ability analysis of the mutants. Mutants were confronted with the phytopathogenic fungi A. alternata (Aa), F. oxysporum (Foc), F. fujikuroi (FFJ) and F. odoratissimum (Foc4) at 28°C for 5 or 10 days in the dark. (PDF) [file pgen.1011282.s008.pdf]

S8 Fig

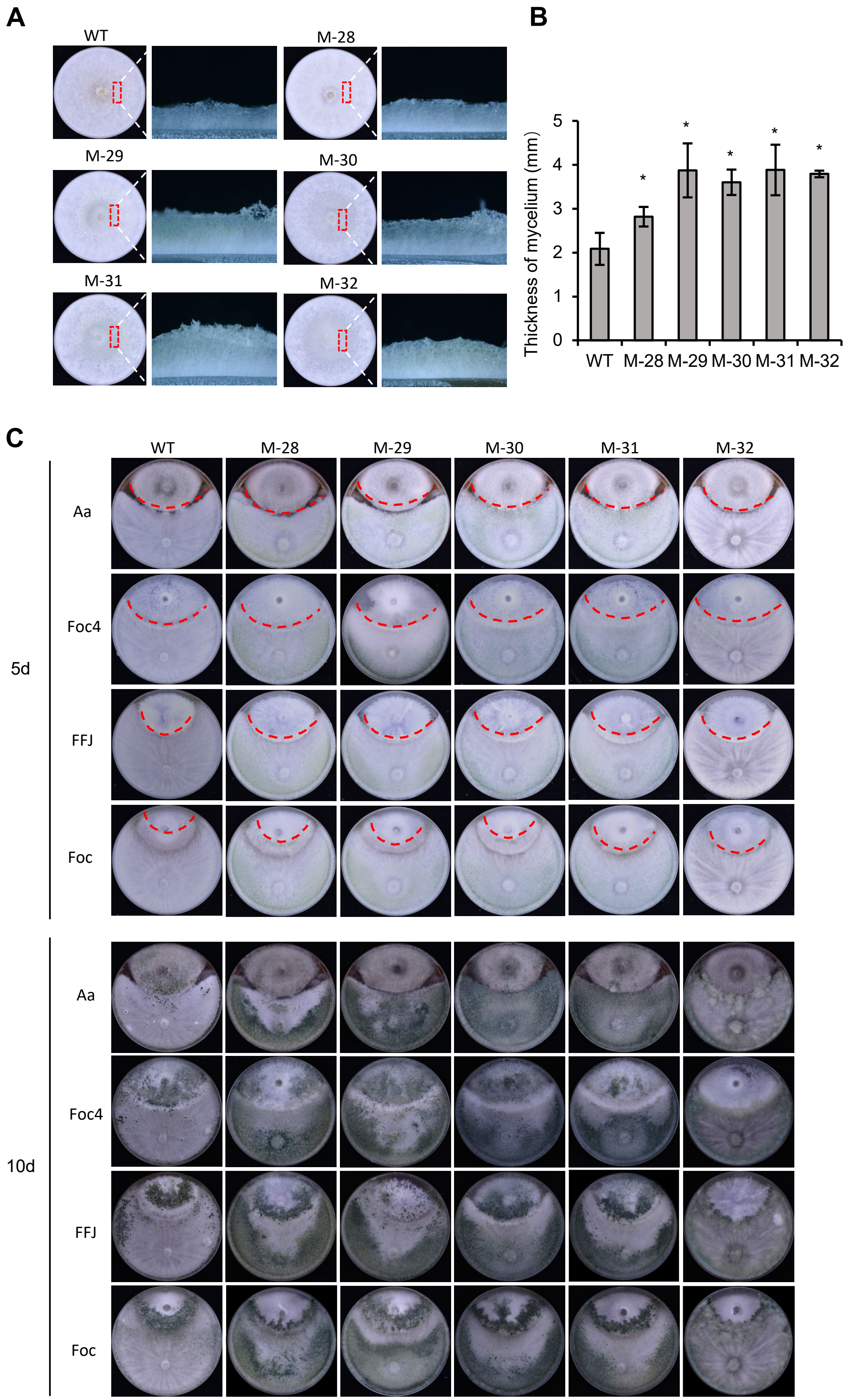

S8 Fig. Phenotype of aerial hyphae and antagonistic activity of five mu-tants screened via UV-mutagenesis.
(A). Phenotype of aerial hyphae of the mutants. (B). Thickness measurement of aerial hyphae in wild type and five mu-tants. Error bar represents the standard deviation (SD) of five biological repli-cates. (C). Antagonistic ability analysis of the mutants. Mutants were confronted with the phytopathogenic fungi A. alternata (Aa), F. oxysporum (Foc), F. fujikuroi (FFJ) and F. odoratissimum (Foc4) at 28 °C for 5 or 10 days in the dark.
